# Co-Evolution of Mitochondrial tRNA Import and Codon Usage Determines Translational Efficiency in the Green Alga *Chlamydomonas*

**DOI:** 10.1371/journal.pgen.1002946

**Published:** 2012-09-20

**Authors:** Thalia Salinas, Francéline Duby, Véronique Larosa, Nadine Coosemans, Nathalie Bonnefoy, Patrick Motte, Laurence Maréchal-Drouard, Claire Remacle

**Affiliations:** 1 Génétique des Microorganismes, Department of Life Sciences, Institute of Botany, University of Li?ge, Li?ge, Belgium; 2 Institut de Biologie Moléculaire des Plantes, UPR 2357, Centre National de la Recherche Scientifique, University of Strasbourg, Strasbourg, France; 3 Centre de Génétique Moléculaire, UPR3404, FRC3115, Centre National de la Recherche Scientifique, Gif-sur-Yvette, France; 4 Functional Genomics and Plant Molecular Imaging, Department of Life Sciences, Institute of Botany, University of Li?ge, Li?ge, Belgium; University of Michigan, United States of America

## Abstract

Mitochondria from diverse phyla, including protozoa, fungi, higher plants, and humans, import tRNAs from the cytosol in order to ensure proper mitochondrial translation. Despite the broad occurrence of this process, our understanding of tRNA import mechanisms is fragmentary, and crucial questions about their regulation remain unanswered. In the unicellular green alga *Chlamydomonas*, a precise correlation was found between the mitochondrial codon usage and the nature and amount of imported tRNAs. This led to the hypothesis that tRNA import might be a dynamic process able to adapt to the mitochondrial genome content. By manipulating the *Chlamydomonas* mitochondrial genome, we introduced point mutations in order to modify its codon usage. We find that the codon usage modification results in reduced levels of mitochondrial translation as well as in subsequent decreased levels and activities of respiratory complexes. These effects are linked to the consequential limitations of the pool of tRNAs in mitochondria. This indicates that tRNA mitochondrial import cannot be rapidly regulated in response to a novel genetic context and thus does not appear to be a dynamic process. It rather suggests that the steady-state levels of imported tRNAs in mitochondria result from a co-evolutive adaptation between the tRNA import mechanism and the requirements of the mitochondrial translation machinery.

## Author Summary

Mitochondria are endosymbiotic organelles involved in diverse fundamental cellular processes. They contain their own genome that encodes a few but essential proteins (e.g. proteins of the respiratory chain complexes). Their synthesis requires functional mitochondrial translational machinery that necessitates a full set of transfer RNAs (tRNAs). As mitochondrial genomes of various organisms do not code for the complete set of tRNA genes, nucleus-encoded tRNAs have to be imported into mitochondria to compensate. Mitochondrial import of tRNAs is highly specific and tailored to the mitochondrial needs. Because transformation of the mitochondrial genome is possible in *Chlamydomonas*, we used this green alga as model to know if a fine regulation of the tRNA import process is possible so that the tRNA population can rapidly adapt to codon usage changes in mitochondria. Here we provide evidence that the regulation of tRNA mitochondrial import process is not dynamic but is rather the result of a co-evolutive process between the import and the mitochondrial codon bias in order to optimize the mitochondrial translation efficiency.

## Introduction

Mitochondria are organelles found in almost all eukaryotic cells [1]. They contain a genetic system that encodes a number of protein-coding genes involved in oxidative phosphorylation, which yields the bulk of the ATP made in cells. The synthesis of these mitochondria-encoded proteins is thus essential for life and requires a complete set of transfer RNAs (tRNAs). In many organisms, however, the number of mitochondrial tRNA genes is not sufficient to ensure mitochondrial translation and nucleus-encoded tRNAs have to be imported from the cytosol to mitochondria. The mitochondrial import of cytosolic tRNAs is a widespread phenomenon and has been experimentally documented in diverse organisms including protozoa, fungi, higher plants and mammals [2]. Interestingly, in some organisms where mitochondrial genomes are apparently equipped with a minimal set of tRNA genes sufficient for mitochondrial translation [3], tRNA import can occur under certain circumstances, as documented in yeast and in human [4], [5]. The number of imported tRNAs into mitochondria ranges from one to the whole set in a species-specific manner [6]–[8].

Our knowledge on the tRNA mitochondrial import regulation is still limited [2], [8]–[10]. The nucleus-encoded tRNAs imported into mitochondria are used in the cytosolic translation machinery and only a small percentage of cytosolic tRNAs is present in mitochondria. Furthermore, variations between the extents of mitochondrial localization of individual nucleus-encoded tRNAs have been observed. Indeed, in *Leishmania tarentolae*, tRNAs are classified into three groups (mainly cytosolic, mainly mitochondrial and shared between the two compartments) according to their relative abundance in the cytosol or mitochondria [11]. In *Trypanosoma brucei*, the quantification of the absolute abundance of each tRNA in the cell and in mitochondria revealed that the extent of their mitochondrial localization fluctuates between 1 and 7.5% [12], [13]. In plants, some evidence supports the idea that a differential distribution of nuclear encoded tRNAs between the cytosol and mitochondria exists. Studies on tobacco tRNA^Gly^ issoacceptors showed that the mitochondria-imported tRNA^Gly^(UCC) represents 2.5% of total tRNA^Gly^(UCC) whereas the mitochondria-imported tRNA^Gly^(CCC) represents 6.5% of total tRNA^Gly^(CCC) [14]. In wheat, the nucleus-encoded tRNA^Leu^(UAA) was shown to be in higher amount in mitochondria than in the cytosol [15] and in potato, the nucleus-encoded tRNAs^Val^ and tRNAs^Thr^ are 2–3 times less abundant that the nucleus-encoded tRNAs^Leu^ [16]. What triggers the differential distribution of nucleus-encoded tRNAs between the cytosol and mitochondria in any of these organisms remain an open question.

In addition it is known that the cellular concentration of the various tRNA species needed for ribosome-dependent protein synthesis correlates well with the respective amounts of amino acids to be incorporated in the newly synthesized proteins [17], [18], in other words correlates with codon usage. In this work we question how such correlation occurs between the mitochondrial import of nucleus-encoded tRNAs and mitochondrial codon usage. To answer this question, the green alga *Chlamydomonas reinhardtii* was used as a model organism. Several reasons dictated this choice. First, its mitochondrial genome only encodes 3 tRNA genes [19] so that its mitochondrial translation machinery primarily depends on tRNA mitochondrial import. Indeed, out of the 49 cytosolic tRNA issoacceptors, 34 are found within mitochondria. Second, as observed in higher plants, this import is highly specific since only necessary cytosolic tRNAs are imported. In addition, the extent of mitochondrial localization for each tRNA species is highly variable. For 31 tRNAs, it ranges from 0.2% to 26% and for three tRNAs it is equal or above 80%. Interestingly, the observed steady-state levels of imported tRNAs correlate with the mitochondrial codon usage, thus suggesting that the levels of imported tRNAs would be defined by the information residing in the mitochondrial DNA in order to allow optimal mitochondrial translation [20]. Third, the mitochondrial genome of *Chlamydomonas* can be manipulated [21] offering the possibility to modify its codon usage.

Given these prerequisites, we decided to replace in *Chlamydomonas* mitochondrial genes an often-used codon by a seldom-used codon encoding the same amino acid and to check the repercussions on tRNA mitochondrial import. For this, the tRNA^Gly^ isoacceptors were used as model and the GGC and GGT Glycine codons were replaced by GGG codons in mitochondrial genes. Our results show that massive changes are not accepted by mitochondria and only two transformants in the homoplasmic state were obtained with 10 and 11 additional GGG codons, respectively. The analysis on the import status of the tRNA^Gly^ isoacceptors showed no adaptation to the new GGG codon content in the mitochondrial genome. We found that these few modifications result in decreased levels and activities of respiratory complexes which could be explained by reduced levels of mitochondrial translation. This demonstrates that the regulation of mitochondrial tRNA import is not dynamic but rather fixed during evolution in order to meet the requirement of the mitochondrial translation machinery.

## Results

### Modification of the mitochondrial glycine codon usage

Three cytosolic tRNA^Gly^ isoacceptors have been identified in *Chlamydomonas*: tRNA^Gly^(CCC), tRNA^Gly^(GCC) and tRNA^Gly^(UCC). The first one, tRNA^Gly^(CCC) is poorly imported into mitochondria and represents 0.15% of total cytosolic tRNA^Gly^(CCC). By contrast, tRNA^Gly^(GCC) and tRNA^Gly^(UCC) are efficiently imported into mitochondria (3.6% and 4.2% of total cytosolic tRNA^Gly^(GCC) and tRNA^Gly^(UCC) respectively) [20]. The tRNA^Gly^(CCC) decodes the GGG codon, a codon only present three times in the whole mitochondrial genome and representing 0.1% of total codons. The tRNA^Gly^(GCC) enables the decoding of both GGC and GGT codons that together represent 7.5% of all the codons in mitochondria [19]. Both tRNA^Gly^(GCC) and tRNA^Gly^(CCC) illustrate the strong correlation observed in *Chlamydomonas* between the efficiency of tRNA import into mitochondria and the codon usage in the mitochondrial genome. So, we decided to replace the GGC and GGT codons by GGG codons in mitochondrial genes in order to increase the needs of tRNA^Gly^(CCC) in mitochondria.

### No transformants can be recovered when a modified version of the *cob* gene containing only GGG codons is used for transformation of the *dum11* mutant

**Figure 1:**
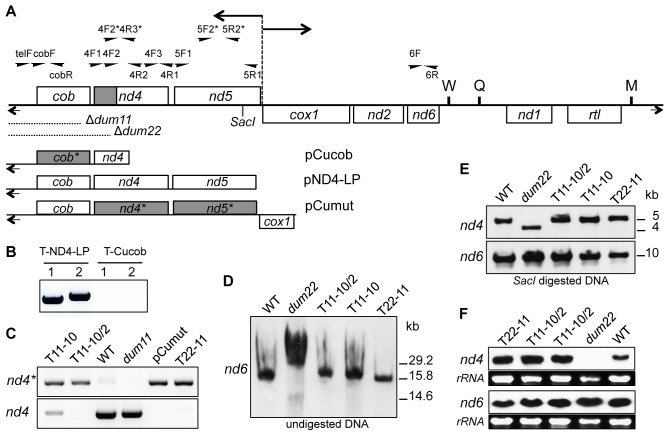
Molecular characterization of the transformants. (A) Schematic map of the *Chlamydomonas reinhardtii* mitochondrial genome. Boxes represent protein-coding genes: (*cob*) apocytochrome *b* of complex III; (*nd1*, *2*, *4*, *5* and *6*) subunits of complex I; (*cox1*) subunit 1 of complex IV; (*rtl*) reverse transcriptase-like protein. W, Q, and M represent tRNAs for Trp, Glu, and Met, respectively. The bidirectional origin of transcription between *nd5* and *cox1* genes is represented by a dashed vertical line and two horizontal arrows. Terminal inverted repeats are shown by short arrows and *Sac*I digestion site at position 5.5 kb (GenBank u03843 numbering) is indicated. Region where modifications on the *nd4* gene were found on T11-10, T11-10/2 and T22-11 transformants is indicated in grey. Position and name of primers are indicated above the map. Primers with a star are specific for the modified gene version (for primer sequence see Table S1). Positions of the *dum11* and *dum22* deletions are shown. Mitochondrial DNA fragments contained in pND4-LP, pCucob and pCumut are schematized. Grey boxes represent the modified genes where GGC/GGT codons were changed in GGG codons. (B) Detection of the *cob* gene in transformants obtained after biolistic transformation with pND4-LP (T-ND4-LP) and pCucob (T-cucob) constructs. PCR analyses were performed with cobF/cobR (1) and telF/cobR (2) pair primers. (C) Detection of the mutated and the wild-type *nd4* genes on T11-10, T11-10/2 and T22-11 transformants. PCR analyses were performed with 4F2*/4R2 and 4F2/4R2 pair primers for the modified *nd4* gene (nd4*) and for wild-type *nd4* gene (*nd4*) respectively. (D–E) Reconstitution of complete mitochondrial genome in T11-10, T11-10/2 and T22-11 transformants. Southern blot analyses were performed (D) on total DNA with the *nd6* PCR probe and (E) on *Sac*I digested DNA with *nd4* and *nd6* PCR probes. (F) Transcript levels of *nd4* and *nd6* genes in T11-10, T11-10/2 and T22-11 transformants. Northern blot analyses were performed on total RNA with *nd4* and *nd6* PCR probes. Loadings of rRNAs are shown.

The pCucob construct that bears the left mitochondrial terminal repeat, a version of the *cob* gene where all the 34 GGT/GGC codons were converted into GGG codons (Figure S1) and 187 bp of the *nd4* gene was used to transform *Chlamydomonas* cells of the *dum11* mutant deleted for part of the *cob* gene and the left terminal repeat (Figure 1A). After a two months selection in heterotrophic conditions (dark+acetate), seven transformants were recovered. PCR analysis using primers specific for the left telomere and the *cob* gene (*i.e*. cobF/cobR and telF/cobR pair primers; Figure 1A) revealed that all of them still bore the deletion of the extremity of the genome, as exemplified for two of them (Figure 1B). Such “transformants” have already been observed previously and likely correspond to mutant cells that survived to the two months period in the dark [21]. In contrast and as expected, when the pND4-LP construct containing a non-mutated version of the *cob* gene was used to transform *dum11* (Figure 1A), 92 true transformants bearing the left telomere and the *cob* gene could be rescued as shown in [22] (Figure 1B).

### Heteroplasmic transformants with up to 10 GGG codons in *nd4* can be selected by transformation of the *dum11* mutant

The rescue of the *cob* gene is essential for the recovery of respiration and the ability to grow in the dark. Results presented above suggest that the presence of GGG codons in the *cob* gene fails to restore growth in the dark. A plausible explanation is that there is not enough of imported tRNA^Gly^(CCC) to allow for an efficient decoding of the novel mitochondrial GGG codons to synthesize COB or in other words that *Chlamydomonas* mitochondrial tRNA import machinery cannot adapt rapidly to a massive change in mitochondrial codon usage. In order to determine whether some adaptation is possible, attempts were made to modify the codon usage in *nd4* and *nd5* genes. These two genes encode subunits of NADH:ubiquinone oxidoreductase (complex I) and mutants deprived for one or the other subunit are still able to grow in the dark, albeit slower than the wild-type strain [23]–[25]. In addition, viable mitochondrial transformants mutated in *nd4* could be recovered after transformation using a transforming DNA bearing a mutation in that gene [21], [22]. A cloned version of the mitochondrial *nd4* and *nd5* genes was thus designed where all the 26 and 55 GGC/GGT codons were replaced by GGG in *nd4* and *nd5* respectively (Figures S2 and S3). The pCumut construct with a mitochondrial DNA fragment comprising the wild-type *cob* gene and the codon modified version of the *nd4* and *nd5* genes was introduced in *Chlamydomonas* cells by biolistic transformation of *dum11* (Figure 1A). After two months in the dark, 559 transformants were recovered. Screening of the transformants for codon modification was based on PCR analysis with four pair primers: two for the *nd4* gene (4F2*/4R2 and 4F1/4R3*) and two for the *nd5* gene (5F1/5R2* and 5F2*/5R1). Each pair of primers is composed of an oligonucleotide specific for mutated positions on the modified gene (indicated with stars) and of an oligonucleotide specific for a non-modified region in the gene (Figure 1A and Figures S2 and S3). After analysis of the 559 transformants by these four PCRs, two transformants (n° 29 and 61) with modifications in the *nd4* gene were found and none for modifications in *nd5* gene. In addition, both transformants were found heteroplasmic as shown for clone 29 (Figure 1C). The exact number of codon modifications, determined by sequencing, is ten in clone 29 and six in clone 61 and all GGG codons were located in the 3′ end of *nd4*, (*i.e.* the closest region with regards to the *dum11* mitochondrial DNA deletion) (Figure 1A). Consequently, the percentage of GGG codons in mitochondria of the two mutants increased from 0.1% in wild type to 0.29% in clone 61 and 0.42% in clone 29. Clone 29 was kept for further analyses and called T11-10. This mutant was also repeatedly subcloned and one year after its isolation, a mutant homoplasmic for the modification of the 10 codons was isolated. This homoplasmic mutant was called T11-10/2 (Figure 1C).

### A homoplasmic transformant with 11 GGG codons in *nd4* can be selected after transformation of the *dum22* mutant deleted for *cob* and *nd4*

As the number of codons modified was low using the *dum11* strain, another recipient strain was then used for transformation: the *dum22* mutant. This mutant strain is deleted for the left terminal repeat, *cob* and the 3′ end of *nd4* gene [21], [22] (Figure 1A). The deletion in *nd4* could favor the integration of homoplasmic codon modification in that gene. The same plasmid as described above (pCumut) was used for transformation. One single transformant could be rescued and sequencing showed that it displayed one more modified codon at the 3′ end of *nd4* gene as compared to the T11-10 transformant (Figure S2). This transformant with 11 modified codons was found to be in the homoplasmic state (Figure 1C) and called T22-11. The percentage of GGG mitochondrial codons increases from 0.1% in wild type strain to 0.45% in T22-11.

To check the integrity of the mitochondrial genome in the three transformants T11-10, T11-10/2 and T22-11, further molecular analyses were performed. Total undigested DNA of each of the three transformants was probed with a mitochondrial *nd6* PCR amplified fragment. All of them displayed the same mitochondrial DNA as the control wild-type strain while the DNA migration profile of the *dum22* recipient strain displayed a different profile due to the presence of deleted monomers and dimers resulting from head to head fusion of deleted monomers, as already noted previously [26] (Figure 1D). In addition, Southern blot experiments on total DNA digested with *Sac*I were performed using two probes, the one cited above that covered *nd6* and the other that covered *nd4*. The three transformants exhibited the same profile as the wild-type strain while the *dum22* strain exhibited a shorter fragment with the *nd4* probe, reflecting the terminal deletion of the genome in that strain (Figure 1E). All these results thus show that the three transformants have recovered a wild-type mitochondrial genome, except for the modification of the codon usage in *nd4*. In addition, Northern blot analysis using the same two probes revealed that *nd4* and *nd6* were expressed at the same level in the three transformants T11-10, T11-10/2 and T22-11 as in wild type (Figure 1F).

### Respiration and growth characteristics of the transformants

To determine whether the mitochondrial codon usage modification either in the heteroplasmic T11-10 or the homoplasmic T11-10/2, T22-11 state would have an impact on physiological activities linked to mitochondria, dark whole cell respiration and doubling times were measured (Table 1). Two wild-type transformants (i.e. transformants having recovered the wild-type mitochondrial sequence after transformation with the pND4-LP construct), one coming from the *dum11* recipient strain T11-WT and one coming from the *dum22* strain T22-WT [22] were added as control strains. No change in the dark respiration rate and no change of the doubling time in heterotrophic condition were observed in the heteroplasmic T11-10 mutant as compared to T11-WT. By contrast, the dark respiration rates of the two transformants homoplasmic for modified codon usage, T11-10/2 and T22-11, are significantly lower than those of their corresponding wild-type transformants (*P*<0.05). In addition, doubling time in heterotrophic condition is significantly higher in both homoplasmic T11-10/2 and T22-11 transformants as compared to T11-WT and T22-WT but not in the light. In conclusion, codon usage modification in *nd4*, when present in the homoplasmic state, impairs respiration and growth of the transformants in the dark.

**Table 1:** Total respiration and doubling time in T11-10, T11-10/2, and T22-11 transformants.

	T11-WT	T11-10	T11-10/2	T22-WT	T22-11
**O_2_ consumption**	30.0±1.0	30.3±1.4	23.5±2.0*	31.7±1.5	17.4±1.6*
**Doubling time (D)**	29.4±1.3	29.1±0.6	44.0±2.3*	29.6±4.4	50.9±10.2*
**Doubling time (L)**	11.2±1.5	11.6±1.0	13.2±1.0	11.0±1.0	12.2±1.0

Dark whole-cell respiratory rates are expressed in nmol of O_2_ min^−1^ 10^−7^ cells ± SD (mean of 3 independent experiments). Doubling times were measured in heterotrophic conditions (D) and mixotrophic conditions (L) and are expressed in hours ± SD (mean of 3 independent experiments). Asterisks indicate statistically significantly differences using Student *t* test with a significance threshold of 0.05.

### Respiratory complexes in transformants with modified codon usage in *nd4*

**Figure 2:**
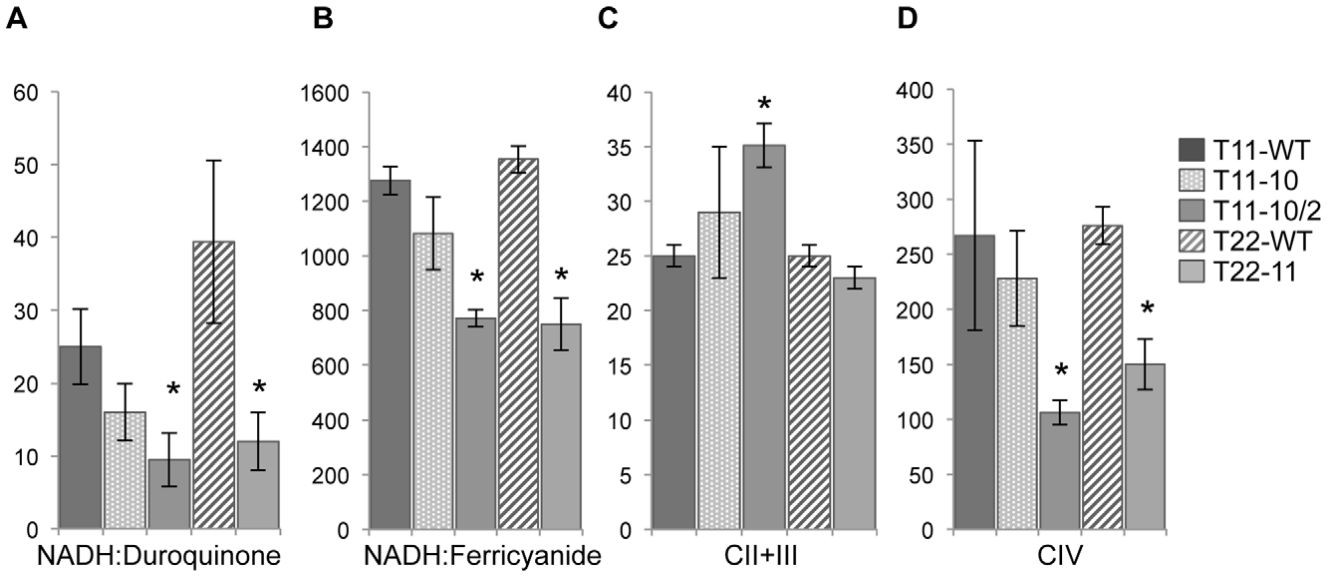
Respiratory enzyme activities of T11-10, T11-10/2, and T22-11 transformants. Respiratory activities were measured on membrane fractions of T11-10, T11-10/2 and T22-11 mutants. NADH:Duroquinone corresponds to the rotenone-sensitive NADH:duroquinone oxidoreductase activity (nmol of NADH oxidized min^−1^ mg of proteins^−1^); NADH:Ferricyanide corresponds to the NADH:Fe(CN)_6_^3−^ oxidoreductase activity (nmol of K3Fe(CN)_6_^3−^ reduced min^−1^ mg of proteins^−1^); CII+III corresponds to the succinate:cytochrome *c* oxidoreductase activity (nmol cytochrome *c* reduced min^−1^ mg of proteins^−1^); CIV corresponds to the cytochrome *c* oxidase activity (nmol of cytochrome *c* oxidized min^−1^ mg of proteins^−1^). Asterisks indicate statistically significantly differences using Student *t* test with a significance threshold of 0.05. Results are means of 3 to 6 independent experiments.

To try to identify the cause of the decreased respiration rates, activities of the respiratory complexes were measured on membrane extracts of the three transformants T11-10, T11-10/2, T22-11. The two wild-type transformants T11-WT and T22-WT were added as control strains (Figure 2). Activity of complex I was measured both at the level of the NADH dehydrogenase activity of the peripheral arm (NADH:ferricyanide activity) and at the level of the whole complex I (oxidation of NADH and subsequent transfer of electrons to the membrane domain: NADH:duroquinone activity). Although there is a decrease of complex I and complex IV activities for the heteroplasmic transformant T11-10 compared to T11-WT, the difference is not significant (*P*>0.05). In contrast, when the codon modification in *nd4* is in the homoplasmic state, a significant 70% decrease (*P*<0.05) of the NADH:duroquinone activity and a significant 45% decrease (*P*<0.05) of the NADH dehydrogenase activity of complex I was noticed in the T11-10/2 and T22-11 transformants. Complex IV is also significantly reduced by about 50% in T11-10/2 and T22-11. In contrast, complex II+III activity was not found modified either in T11-10 or in T22-11 but was found significantly increased in T11-10/2. This increase in complex II+III activity is usually observed in *Chlamydomonas* mutants deprived of complex I activity and could represent a kind of compensatory effect [22], [23].

The decreased activities of respiratory complex I and IV can thus explain why reduced respiration rates are found in the homoplasmic transformants. In addition, we can conclude that we are able to distinguish the impact of the codon modification as far as the codon modification is found in the homoplasmic state (T11-10/2 and T22-11), whatever the strain used for mitochondrial transformation (*dum11* or *dum22*). In contrast, no differences could be detected in the heteroplasmic T11-10 transformant. This latter strain contains a mixture of wild-type and mutant copies, it is therefore likely that the impact of the codon modification is hidden by the wild type copies of the mitochondrial genome. As the two homoplasmic transformants show very similar profile, we kept only the T22-11 transformant for further analyses because it contains the highest number of modified GGG codons (11).

**Figure 3:**
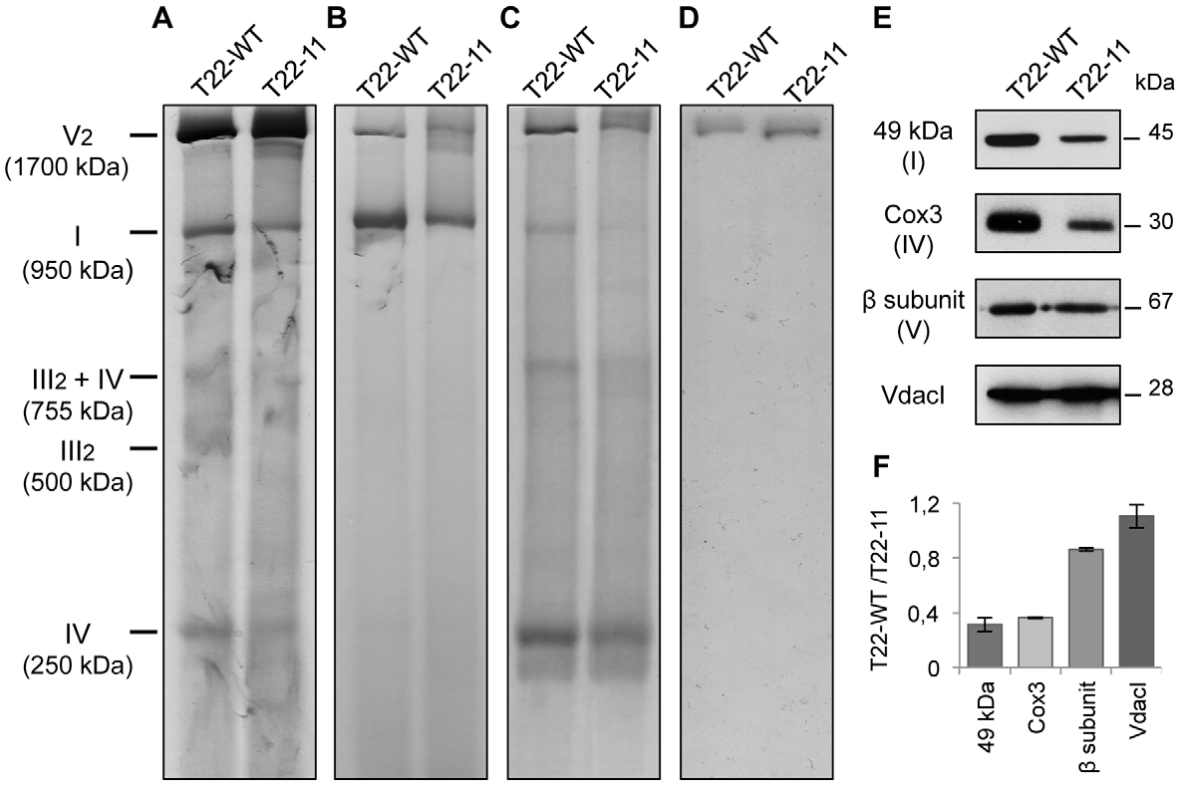
Analysis of mitochondrial complexes of T22-11 transformant. Solubilized mitochondria (30 µg) were loaded on a BN-PAGE and stained (A) with Coomassie Blue, (B) for the NADH dehydrogenase activity of complex I, (C) for the complex IV activity and (D) for the ATP synthase activity. (E) Mitochondrial proteins of T22-WT and T22-11 (10 µg) were separated by SDS-PAGE, blotted and probed with antisera against the 49 kDa subunit of *Chlamydomonas* complex I, against the β subunit of the *Chlamydomonas* mitochondrial ATP synthase, against the Cox3 subunit of *S. cerevisiae* and against the VdacI *Chlamydomonas* protein. (F) Densitometry analysis of the Western blots. Results are means of 2 independent experiments.

To analyze the assembly of the respiratory complexes, mitochondria isolated from T22-WT or T22-11 cell-wall less strains were analyzed on Blue Native PAGE (BN-PAGE). For that purpose, equal amounts of mitochondrial proteins of both strains were solubilized with *n*-dodecyl-β-D-maltoside. Complex I was detected by two stainings: Coomassie Blue staining (Figure 3A) which detects the respiratory complexes and NADH/NBT (nitroblue tetrazolium) staining (Figure 3B) which reveals the NADH dehydrogenase activity of complex I. Complex I was detected at 950 kDa in both T22-WT and T22-11, demonstrating that the codon modification in *nd4* does not prevent the assembly of the whole complex (Figure 3A and 3B). However, both Coomassie Blue and NADH/NBT stainings of complex I are decreased in T22-11. Complex IV was detected at 250 kDa (Figure 3A). Again, both the activity (Figure 3C) and the amount (Figure 3A) are similarly reduced. Dimeric complex V was detected at 1700 kDa (Figure 3A). Contrary to the situation observed for the other complexes, no clear modification of amount (Figure 3A) or activity (Figure 3D) could be seen.

We then analyzed the steady-state levels of various mitochondrial proteins by SDS-PAGE and Western blotting (Figure 3E). The 49 kDa subunit of complex I and the Cox3 subunit of complex IV showed a strong decrease of their amount in T22-11 as compared to T22-WT. In contrast, the steady-state level of the β subunit of complex V was not significantly affected. As a control, a porin from the outer mitochondrial membrane (VdacI) did not present any modification. Densitometry analysis of the Western blots confirmed these observations (Figure 3F).

Altogether, these results suggest that while neither components of the outer membrane, such as VdacI, nor complex V of the respiratory chain are affected by modification of mitochondrial codon usage, there is a clear decrease in both the amount and the activity of respiratory complexes I and IV in the homoplasmic mutant.

### Membrane potential is not affected in T22-11 mitochondria

**Figure 4:**
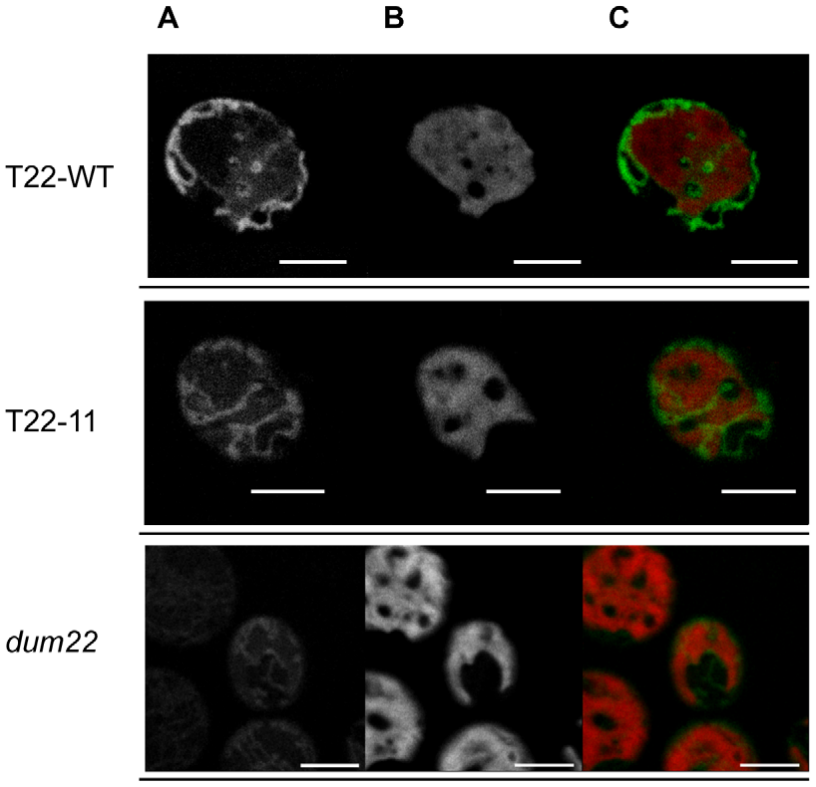
Confocal microscopy of mitochondria from T22-WT, T22-11, and *dum22* stained with MitoTracker dyes. Visualization in T22-WT, T22-11 and *dum22* strains (A) of mitochondria by MitoTracker dye, (B) of chloroplast by chlorophyll autofluorescence and (C) of the overlapping images in *Chlamydomonas* cells. The white line corresponds to 5 µm.

Complex I links the electron transfer from NADH to ubiquinone to the pumping of four protons from the matrix into the intermembrane space. Similarly, complex IV links the electron transfer from cytochrome *c* to molecular oxygen to the pumping of four protons across the inner membrane. To see whether membrane potential of mitochondrial membranes could be affected by the reduced activity and assembly of complex I and IV in T22-11 compared to T22-WT, mitochondria from living cells of these transformants were labeled separately with a MitoTracker dye and observed under confocal laser microscopy. The MitoTracker used (MitoTracker Orange CMTMRos) is sequestered in the mitochondria when entering an actively respiring cell. It is thus dependent on membrane potential. Figure 4 illustrates the analysis of T22-11, T22-WT and *dum22*, the recipient strain of the transformation. Mitochondria of this latter mutant are completely deprived of both complex I and complex III assembly and activity. As expected, labeling of the *dum22* mitochondria is very weak as compared to the T22-WT strain, meaning that membrane potential is affected in this recipient strain. In contrast, there was no obvious difference of mitochondria labeling between T22-WT and T22-11 suggesting that *in vivo*, the impact of the reduction of the complex I and complex IV activities on membrane potential is minor.

### Steady-state levels of mitochondrial tRNAs are marginally modified in the T22-11 transformant

**Figure 5:**
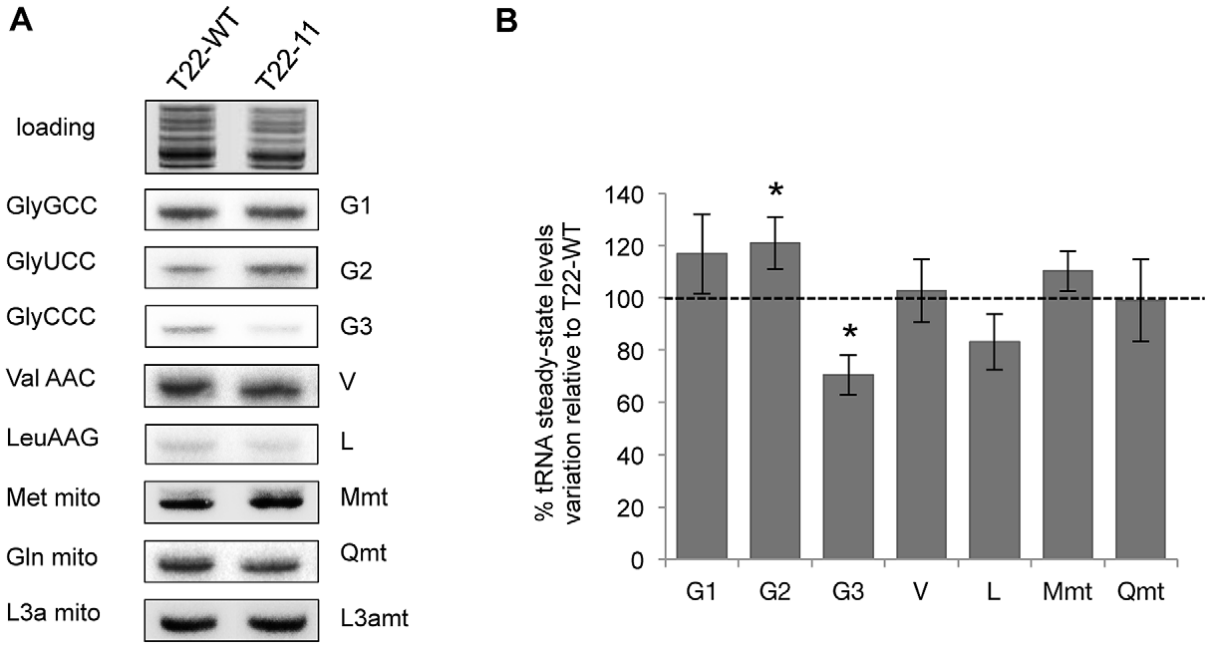
Analysis of the import status of mitochondrial tRNAs in T22-11 transformant. (A) Northern blot analysis of mitochondrial tRNAs extracted from the T22-WT strain and T22-11 transformant. Hybridizations were performed with radiolabeled oligonucleotides specific for cytosolic tRNA^Gly^(GCC) (G1), tRNA^Gly^(UCC) (G2), tRNA^Gly^(CCC) (G3), tRNA^Val^(AAC) (V) and tRNA^Leu^(AAG) (L); for mitochondrial tRNA^Met^ (M mt), tRNA^Gln^ (Q mt) and for the mitochondrial L3a rRNA (L3a mt). (B) Signals were quantified and normalized with the L3a mt signal. Results are the means of 3 to 5 independent experiments and correspond to the percentage of variation of tRNA steady-state levels in the T22-11 transformant as compared to the T22-WT strain. Asterisks indicate statistically significant differences using Student *t* test with a significance threshold of 0.05.

Respiratory complexes for which levels or activities were decreased all contain mitochondria-encoded subunits: complex I contains five mitochondria-encoded subunits (ND1, 2, 4, 5 and 6) and complex IV contains one subunit (COX1). In contrast, complex V does not contain any mitochondria-encoded subunits. This suggests that mitochondrial translation could be affected in the transformant and led us to analyze the steady-state levels of imported and non-imported tRNAs in T22-11 mitochondria. For that purpose, Northern blot experiments on mitochondrial tRNAs extracted from the T22-WT and the T22-11 transformants were performed. A probe directed against the 3La mitochondrial rRNA was used to normalize the signals obtained with the probes specific for seven different tRNAs (Figure 5A and Table S2). Since the codon modifications in T22-11 increase the number of GGG codons, we first focused on the steady-state levels of the tRNA^Gly^(CCC). According to our hypothesis, if a fine-tuning of tRNA import exists to rapidly adapt the tRNA population to the needs of *Chlamydomonas* mitochondria, then an increased amount of mitochondrial tRNA^Gly^(CCC) is expected in T22-11 as compared to T22-WT as a result of an increased tRNA import efficiency. Rather, a 30% decrease of its steady-state level was observed (Figure 5B). For the two other tRNA^Gly^ isoacceptors namely tRNA^Gly^(GCC) that recognizes GGC/GGT codons and tRNA^Gly^(UCC) that recognizes the GGA codon, a 17% and a 21% increase of their steady-state levels were respectively observed in mitochondria . The analysis of the steady-state levels of two mitochondrial tRNAs, tRNA^Met^ and tRNA^Gln^, showed no significant difference (*P*>0.05) indicating that mitochondrial gene expression is not affected in T22-11. Finally, the levels of two other cytosolic tRNAs were analyzed, i.e. of tRNA^Val^(AAC) that is imported into mitochondria and of tRNA^Leu^(AAG) that is mostly not imported [20]. The steady-state level of tRNA^Val^(AAC) is not affected while a diminution of 17% of the steady-state level of tRNA^Leu^(AAG) was observed. Since in wild-type strain, the mitochondrial level of tRNA^Leu^(AAG) is comparable to the background of contamination [20], this decrease (17%) may just reflect a lower cytosolic contamination of purified mitochondria from T22-11 as compared to T22-WT. This may also explain why we observed a similar decreased level for tRNA^Gly^(CCC) which, as tRNA^Leu^(AAG), is present at a very low level in mitochondria.

In conclusion, although a slight increase of the steady-state levels of the two other tRNA^Gly^ isoacceptors was observed, which could reflect some flexibility of tRNA mitochondrial import, the increase of GGG codons from 3 (found in the *rtl* gene) in the T22-WT transformant to 14 in the T22-11 transformant does not enhance the mitochondrial import of nucleus-encoded tRNA^Gly^(CCC). Taking as a whole, this analysis strongly suggests that tRNA mitochondrial import cannot rapidly adapt to fast changes induced in the mitochondrial genome.

### *In organello* protein synthesis is less efficient in T22-11 mitochondria

**Figure 6:**
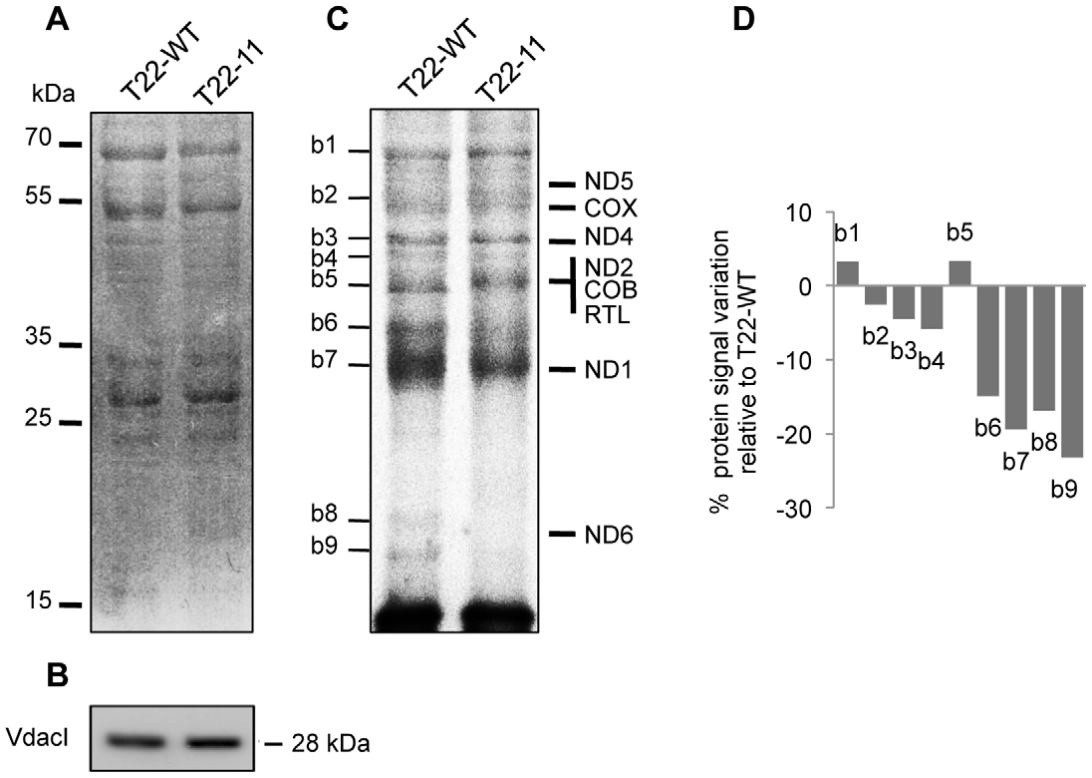
Mitochondrial *in organello* protein synthesis of T22-11 transformant. (A) Mitochondrial proteins of T22-WT and T22-11 (25 µg) were loaded on SDS-PAGE after mitochondrial *in organello* protein synthesis and stained with Coomassie Blue. (B) Mitochondrial protein samples (10 µg) coming from the same experiment as described in (A) were separated by SDS-PAGE, blotted and probed with antisera against the VdacI *Chlamydomonas* protein. (C) Mitochondrial translated proteins from experiment (A) were visualized by autoradiography. Expected migration of the eight mitochondrial proteins are indicated. Major bands obtained in the *in organello* protein synthesis are indicated from b1 to b9. (D) Major bands (b1 to b9) were quantified. The histogram corresponds to the percentage of variation in the T22-11 transformant as compared to the T22-WT strain. The experiment was repeated two times and showed the same decrease of the annotated bands.

The *nd4* gene that codes for one subunit of respiratory complex I is the only gene modified in the T22-11 mutant. Surprisingly, this transformant shows a diminution of the level and the activity not only of complex I but also of complex IV, two complexes that contain mitochondria-encoded subunits. As we have demonstrated above that the steady-state level of the tRNA^Gly^(CCC) cannot adapt to the modification of codon usage in mitochondria and as codon bias can affect translation [27], we decided to compare the *in organello* protein synthesis efficiency of mitochondria isolated from the T22-11 and T22-WT transformants. After incubation in the presence of ^35^S-Methionine under conditions optimized for higher plant *in organell*o protein synthesis, proteins were extracted from mitochondria and analyzed by PAGE. Coomassie Blue staining (Figure 6A) was used as a loading control. The use of equal amount of purified mitochondria was also attested by Western blot analysis using an antibody raised against the mitochondrial VdacI (Figure 6B). The radiolabeled synthesized proteins were visualized by autoradiography of the Coomassie Blue stained gel (Figure 6C). The expected migration of the eight proteins encoded by the *Chlamydomonas* mitochondrial DNA is indicated according to their theoretical molecular weights. For both transformants, nine major bands (annotated b1 to b9) are visible upon autoradiography (Figure 6C). To our knowledge, there is no report yet of *in organello* protein synthesis data in *Chlamydomonas* mitochondria and more studies will be necessary for a detailed investigation of the pattern. However, the fact that only a small number of bands were detected makes us confident that we are truly observing the translation of mitochondria-encoded proteins. In addition, even if we observed some discrepancies between the theoretical migration profile of the eight mitochondria-encoded proteins and the experimental protein pattern resulting from *in organello* synthesis, we can conclude that the amount of the majority of the synthesized proteins was decreased in T22-11 and this was confirmed by quantification of the signals (Figure 6D). This is especially true for b8 and b9, which could correspond to isoforms of ND6, the smallest protein encoded by the mitochondrial genome, whose low molecular weight allows us to discriminate it from the other mitochondria-encoded proteins. There were only two exceptions, b1 and b5, which showed a slight upregulation. However, the apparent molecular weight of the protein corresponding to b1 is higher than that expected for a mitochondria-encoded protein, thus suggesting that this protein is likely a plastidial or cytosolic contaminant. Altogether, these results show that the modification of codon usage in *nd4* has an impact on the translation efficiency of the whole mitochondrial genome, which could explain why the amount of the respiratory complexes containing mitochondria-encoded subunits (complex I and IV) is decreased.

## Discussion

We have tested here the idea of an adaptive tRNA import process in mitochondria. Different mitochondrial transformations were performed in order to replace often-used codons (i.e. GGC/GGT) by a seldom-used codon (i.e. GGG codon). Only three transformants were obtained with a maximum of 11 modifications in the *nd4* gene. Like previously observed [22], the efficiency of transformation was very low using the *dum22* mutant, since only one transformant (T22-11) was recovered. However, this transformant was very useful because it was the only one to be homoplasmic directly after the two-month selection in the dark and it contained the highest number of GGG codons. It thus appears that the mitochondrial genome of the transformant integrated the minimal sequence to recover a non-deleted genome, which included GGG codons. In contrast, when it is possible to avoid the integration of the modified codons, for example when the *dum11* mutant was used as recipient strain, a much higher number of transformants (559) were recovered. However, amongst them, a very few number (2 out 559) had GGG codons and in that case, they were in the heteroplasmic state. Nevertheless, homoplasmic state could be reached after one year of repeated rounds of subcloning. This strongly suggests that the integration of modified codons in the mitochondrial DNA is unfavorable for the *Chlamydomonas* cells. Indeed, homoplasmic transformants presented a reduced growth rate and respiration and longer doubling time in heterotrophic conditions whereas their growth rates were not affected in mixotrophic conditions (light + acetate) probably because in that case, growth relies on photosynthesis. *In vitro*, they exhibited reduced respiratory enzymes activities and reduced amounts of complexes I and IV. *In vivo*, mitochondrial membrane potential did not seem modified. This suggests that proton pumping of complex I and complex IV are not severely affected or that the increase in complex II+III activity could compensate for the less efficient proton pumping at complex I and IV. Similar results were already observed for complex I mutants deprived of complex I activity and assembly [22]. The impossibility to obtain transformants with more codon modifications indicates that the number of modifications cannot exceed a particular threshold without being lethal in our conditions of selection. Thus, the tRNA mitochondrial import is not dynamically regulated but rather has been fixed during evolution in order to meet the requirement of the mitochondrial translation machinery. The analysis of the T22-11 transformant harboring 11 GGC/GGT codons modified into GGG codons in *nd4* showed that the tRNA^Gly^(CCC) import was not adapted to the new GGG codon content in the mitochondrial genome. We cannot exclude the possibility that the inability to increase the import of tRNA^Gly^(CCC) into mitochondria comes from the unavailability of this tRNA in the cytosol. Indeed, analyzing mitochondrial and cytosolic codon frequencies, we previously showed that tRNA^Gly^(CCC) is more solicited in the cytosol than in mitochondria. As tRNA^Gly^(CCC) is encoded by a single gene [28] and as cytosolic tRNA levels are well tuned to cytosolic translational demand, the absence of adaptation for the new need of tRNA^Gly^(CCC) in mitochondria of transformants may be due to insufficient amount of free tRNA^Gly^(CCC) that are engaged in the cytosolic translational machinery. Further studies aiming at adding several tRNA^Gly^(CCC) gene copies in the nuclear genome of the T22-11 transformant, thus increasing the steady-state level of this tRNA in the cytosol, must tell us whether the overexpressed tRNA can compensate for the new need of extra tRNA^Gly^(CCC) in mitochondria of this mutant strain or whether its mitochondrial import is still restricted by the import machinery.

Yet, a slight increase of the import of the two other tRNA^Gly^ isoacceptors was observed. An attractive hypothesis to explain this would be that this increase would compensate the shortage of tRNA^Gly^(CCC). Indeed, depending on the repertoire of isoacceptor tRNAs and on the type of modified nucleotides found at the first position of the anticodon, four main decoding strategies were identified by Grosjean et al. [29]. Depending on the strategy used, the GGG codon can also be read by a tRNA^Gly^(UCC), meaning that in theory two isoacceptor tRNAs can read the same codon. However, in none of the strategy, the tRNA^Gly^(GCC) would be able to read GGG codons. In *Chlamydomonas* mitochondria, the most abundant tRNA^Gly^ has a GCC anticodon, and the tRNA^Gly^(UCC) is found in much lower amount [20]. Indeed, there are 17 tRNA^Gly^(GCC) gene copies but only one gene for tRNA^Gly^(UCC) and one for tRNA^Gly^(CCC) in the nuclear genome [28]. Considering that the most abundant tRNA^Gly^, the tRNA^Gly^(GCC), cannot compete for decoding the GGG codons and that the one that can potentially compete, the tRNA^Gly^(UCC), is present in low amount, it is likely that the other tRNA^Gly^ present in mitochondria cannot compensate the even modest increase in GGG codon content in the mutant strains. Furthermore, it has been demonstrated in chloroplasts that the tRNA^Gly^(UCC) is capable of reading the four Glycine codons according to the superwobbling rule [30]. However supperwobble is only possible if U34 is not modified and nucleus-encoded imported tRNA^Gly^(UCC) was shown to be post-transcriptionally modified at this position in plants [31], [32]. Thus, although we cannot completely exclude that the imported mitochondrial tRNA^Gly^(UCC) can be used as an alternative to compensate for the inability to rapidly increase the import level of the tRNA^Gly^(CCC) in order to read the additional GGG codons, it is very unlikely.

We have thus in our hand a mutant affected for mitochondrial codon usage in *nd4*. Interestingly, the consequences of these modifications are not restricted to the multisubunit enzyme (complex I) which comprises ND4 but also concern other respiratory complexes such as complex IV which contains a mitochondria-encoded subunit (COX1). In contrast, complex V that does not bear any mitochondria-encoded subunit in *Chlamydomonas* is not (or much less) affected as well as VdacI, a porin of the outer mitochondrial membrane. The general reduction of respiratory enzymes containing mitochondria-encoded subunits could be explained by the decrease of mitochondrial translation that we detected by *in organello* protein synthesis. The question of the reason why the modified codon usage in *nd4* affects the whole mitochondrial translation process and not only that of ND4 is open. The recent work in the yeast *Saccharomyces cerevisiae* could shed light on that point [27]. These authors showed that translational efficiency is optimized by a mechanism that relies on proportional use of codons according to their cognate tRNA concentrations, suggesting that the codon-tRNA balance is the major factor determining translation efficiency [27]. Importantly, they propose that preferentially used codons are not translated faster than unpreferred ones but that this phenomenon is a result of codon usage in proportion to cognate tRNA concentrations, the optimal strategy in enhancing translational efficiency under tRNA shortage. According to their model, the introduction of eleven GGG codons in the *nd4* gene would break the established codon-tRNA balance causing the decrease of translational efficiency in mitochondria, and indeed, this is what was observed for the T22-11 transformant. To our knowledge, this is the first time that the impact of codon bias on translation by itself is demonstrated in mitochondria. This explains why the fitness of the cells is decreased in conditions where growth relies on respiration (heterotrophic conditions).

In conclusion, our work shows that mitochondrial tRNA import cannot adapt rapidly in *Chlamydomonas* and that codon bias has a direct effect on translation efficiency. These data demonstrate that the information residing in the mitochondrial DNA does not regulate tRNA import. So the fine-tuning observed in *Chlamydomonas* mitochondria between tRNA import and the codon usage appear to originate from a co-evolution process rather from a dynamic adaptation of cytosolic tRNA import into mitochondria. Future work on the understanding of how this co-evolution works in plants should focus on the characterization of the tRNA import machinery and more precisely on the first stages of tRNA import (i.e. during their targeting from the nucleus to the mitochondrial surface). This would undoubtedly give a more comprehensive picture of how tRNA import regulation into mitochondria is achieved.

## Materials and Methods

### Strains and growth conditions

The following mitochondrial mutants of *C. reinhardtii* were used as recipient for the biolistic transformation: *dum11* that exhibits a 1.2 kb deletion extending beyond codon 147 of *cob* and responsible for loss of complex III activity and the *dum22* mutant possessing a deletion extending beyond the 3′end of *nd4* sequence and responsible for loss of complex I and III activity. Cells were routinely grown at 25°C under heterotrophic (dark + acetate) or mixotrophic (light + acetate) conditions. Light conditions were 50 µE m^−2^ s^−1^ and Tris-acetate phosphate (TAP) medium [33] was used.

### Plasmids used for *Chlamydomonas* mitochondrial transformation

The pND4-LP, the pCucob and the pCumut constructs were purchased from ATG-biosynthetics Company (https://www.atg-biosynthetics.com/). The pND4-LP construct corresponds to the first 4966 bp of the mitochondrial genome, including the left telomere, the *cob* gene, the *nd4* gene and the *nd5* gene [22]. The pCucob construct corresponds to the first 1900 bp of the mitochondrial genome, including the left telomere, the mutated version of *cob* gene and 187 bp of the *nd4* gene cloned into *Eco*RV*/Spe*I digested pUC57 vector. The pCumut construct corresponds to the 5400 bp of the mitochondrial genome, including the left telomere, the *cob* gene, the mutated version of *nd4* gene, the mutated version of the *nd5* gene and 335 bp of the *cox1* gene cloned into *Eco*RI*/Pst*I digested pSB3C5 vector. The mutated version of *cob* gene, *nd4* gene and *nd5* gene in pCucob and pCumut constructs correspond to genes in which the GGC/GGT codons were replaced by GGG codons. For mitochondrial transformation, pND4-LP, pCucob and pCumut constructs were linearized by *Bgl*I, *Dra*I, *Pvu*II enzymes respectively.

### Mitochondrial transformation procedures

Cells were grown in liquid TAP medium up to exponential phase (2–3×10^6^ cells) and spread at high density on TAP plates (10^8^ cells per plate). Plates were bombarded with tungsten beads coated with linearized DNA at a concentration of 1 µg/µL by using a Bio-Rad PDS-1000He apparatus under a pressure of 1,100 psi and a partial vacuum in the chamber corresponding to a reading of at least 29 inches Hg, according to [21].

### DNA, RNA, and PCR analyses

*C. reinhardtii* total nucleic acids were prepared according to [34]. For Southern blot analyses, total DNA (10 µg) was digested by *Sac*I enzyme, separated on 0.8% agarose gel and transferred onto Hybond-N^+^ membrane (Amersham Pharmacia Biotech). For Northern blot analyses, total RNA (15 µg) was separated on 0.8% agarose-formaldehyde gel and transferred onto Hybond-N^+^ membrane. Digoxigenin-labeled PCR products of cDNA fragments were used as gene probes and detected with anti-digoxigenin-AP conjugates and CSPD as substrate (Roche Molecular Biology). For PCR analyses, amplification was made either with total DNA or directly with *Chlamydomonas* colonies according to a protocol derived from [35]. Sequencing was performed directly on amplified products by Beckman Coulter Genomics (Essex, UK).

### Whole-cell respiration

Dark respiration rates of cells that were grown mixotrophically i.e. TAP medium in the light, were measured using a Clark electrode (Hansatech Instruments, King's Lynn, England) as described in [26].

### Enzyme activities

Enzyme activity analyses were performed on membrane fractions prepared as described in [23]. NADH:ferricyanide oxidoreductase, complex I (rotenone-sensitive NADH:duroquinone oxidoreductase), complex II + III (succinate:cytochrome *c* oxidoreductase), and complex IV (cytochrome *c* oxidase) activities were measured following published procedures [23], [24].

### Purification of mitochondria

Crude mitochondrial fractions were isolated from cell wall-less *Chlamydomonas* T22-WT and T22-11 strains by digitonin treatment according to [24]. The mitochondrial fraction was then loaded on a discontinuous Percoll gradient (13%/21%/45%). Purified mitochondria were recovered at the 45/21 interface and washed two times in MET buffer containing 280 mM Mannitol, 10 mM Tris–HCl pH 7, 0.5 mM EDTA and 0.1% BSA by 10 min centrifugation at 11000 g.

### Protein complex analysis

Protein complex analyses were conducted on purified mitochondria and the protein content was determined according to [36].

To conduct blue native polyacrylamide gel electrophoresis (BN-PAGE) analyses, protein complexes from purified mitochondria were solubilized in the presence of 1.5% (weight/volume) *n*-dodecyl-β-D-maltoside, 375 mM 6-aminohexanoic acid, 250 mM EDTA and 25 mM Bis-Tris pH 7.0. Solubilized protein complexes were centrifuged for 20 min at 15000 g at 4°C to remove insoluble matters. One percent (weight/volume) of Coomassie Blue G was then added to the supernatant prior to separation by electrophoresis on a 4 to 12% acrylamide gradient BN gel [37]. Coomassie Blue staining and in-gel detection of NADH dehydrogenase, ATP synthase or complex IV activities were performed as described in [23]. SDS-PAGE was performed according to standard protocols. Polyclonal antibodies directed against the *Chlamydomonas* 49 kDa subunit (1∶3000), against the *Chlamydomonas* β subunit of the mitochondrial ATP synthase (1∶150000), against the Cox3 subunit of *S. cerevisiae* (1∶1000) and against the *Chlamydomonas* VdacI protein (1∶20000) were used for Western blotting analyses.

### MitoTracker staining and confocal microscopy

Synchronized cells (1.10^8^) were washed once with TAP medium then incubated in the dark for 30 min in TAP medium added with 1 µM of MitoTracker Orange CMTMRos (Molecular Probes, Leiden, The Netherlands). Stained cells were washed with TAP medium and living cells were directly observed on Superfrost plus slide (Menzel-Glaser, Braunschweig, Germany). For living cell imaging, a Leica TCS SP5-II AOBS inverted confocal laser microscope (Leica Microsystems) and a 63×1.2 numerical aperture Plan-Apo water-immersion objective were used to collect images at 1024×1024 pixel resolution. MitoTracker Orange was detected by using an excitation wavelength of 543 and the fluorescence emission was dispersed and recorded at 550–590 nm. The autofluorescence of chlorophyll was detected by using an excitation wavelength of 488 and the fluorescence emission was dispersed and recorded at 650–750 nm. The diameter of the pinhole was set equal to the Airy unit, and we ensured that the maximal fluorescence signal was not saturating the photomultiplier tubes. A series of optical sections were taken to analyze the spatial distribution of mitochondria, they were recorded with a Z-step of 0.5 µm.

### Northern analysis and quantitation of tRNA import

Mitochondrial tRNAs were extracted from the T22-WT and T22-11 strains according to [20]. Mitochondrial tRNAs (1.5 µg) were fractionated by polyacrylamide gel electrophoresis and transferred onto Hybond-N^+^ membrane (Amersham Pharmacia Biotech). For hybridizations, radiolabeled tRNA specific oligonucleotides were used as probes (Table S1). Hybridization and washing were performed as described in [20]. For each specific probe, signals detected with a FLA-7000 phosphor imager (Fujifilm) were quantified using the software ImageGauge (Fujifilm).

### *In organello* protein synthesis

*In organello* protein synthesis experiments were performed as described in [38]. One hundred µg of mitochondrial proteins were resuspended in a solution containing 5 mM KH_2_PO_4_, pH 7.0, 300 mM Mannitol, 60 mM KCl, 50 mM Hepes, 10 mM MgCl_2_, 10 mM Na-malate, 10 mM Na-pyruvate, 2 mM GTP, 2 mM DTT, 4 mM ADP, 0.1% (w/v) BSA, 25 µM of an unlabeled 19-amino acid mix solution, and 30 µCi (>1000 Ci/mmol ^35^S-Methionine). Reactions were performed in 100 µL for 60 min at 25°C with gentle shaking. The reaction was stopped by the addition of 1 mL MET buffer containing 10 mM of Methionine. After 5 min centrifugation at 11000 g, the mitochondrial pellet was analyzed on SDS-PAGE.

## Supporting Information

Figure S1 Alignment of the wild-type and the modified genes. Modified nucleic acids are indicated in white.
(PDF)

Figure S2 Alignment of the wild-type and the modified *nd4* gene. Modified nucleic acids are indicated in white. Position and name of the oligonucleotides specific for modified *nd4* gene are indicated by a long arrow. The ten modified GGC/GGT codons into GGG codons in the T11-10 transformant are framed in grey. The additional codon found in the T22-11 transformant is framed in black.
(PDF)

Figure S3 Alignment of the wild-type and the modified *nd5* gene. Modified nucleic acids are indicated in white. Position and name of oligonucleotides specific for modified *nd5* gene are indicated by a long arrow.
(PDF)

Table S1 Oligonucleotides used for molecular characterization of the transformants. Position indicates the location of the oligonucleotide in the *Chlamydomonas* mitochondrial genome according to the GenBank u03843 numbering.
(PDF)

Table S2 Oligonucleotides used for Northern analysis on mitochondrial tRNA fractions.
(PDF)
