# Correction: Co-Evolution of Mitochondrial tRNA Import and Codon Usage Determines Translational Efficiency in the Green Alga *Chlamydomonas*

**DOI:** 10.1371/journal.pgen.1008719

**Published:** 2020-03-31

**Authors:** Thalia Salinas, Francéline Duby, Véronique Larosa, Nadine Coosemans, Nathalie Bonnefoy, Patrick Motte, Laurence Maréchal-Drouard, Claire Remacle

After this article [1] was published, questions were raised about possible vertical discontinuities between lanes in Figs 1B, 1D and 5A. The original image data supporting these figures are provided here in S1 File.

All data reported in Fig 1B are from the same gel, but there is a splicing junction between the “T-ND4-LP” and “T-Cucob” results where lanes from the original gel were removed in preparing the figure. The authors have provided an updated version of Fig 1 and its legend here. In the updated Fig 1B, the image splicing junction is indicated by a blank area and the separated panels represent fragments of the same gel image (see S1 File).

As shown in S1 File, Fig 1D reflects contiguous data from the original image.

In Fig 5A, each panel reports data from the same exposure of the same northern blot, but for all panels except the Val AAC panel there are image splicing junctions between lanes 1 and 2, where cytosolic data from the original northern blots were removed in preparing the figure. The authors have provided an updated version of Fig 5 here. In the updated Fig 5A, image splicing junctions are indicated by blank area between lanes, and the G2, L, and Qmt panels have been replaced due to the unavailability of the raw image data for the original panels. The updates to Fig 5A do not affect the quantification shown in Fig 5B. Quantitative data supporting Fig 5B are in S2 File.

Raw data underlying all other results reported in the article are available upon request from the corresponding author, except for four phosphorimager images that are not accessible anymore.

The authors apologize for not indicating the splicing junctions clearly in the published article.

**Fig 1:**
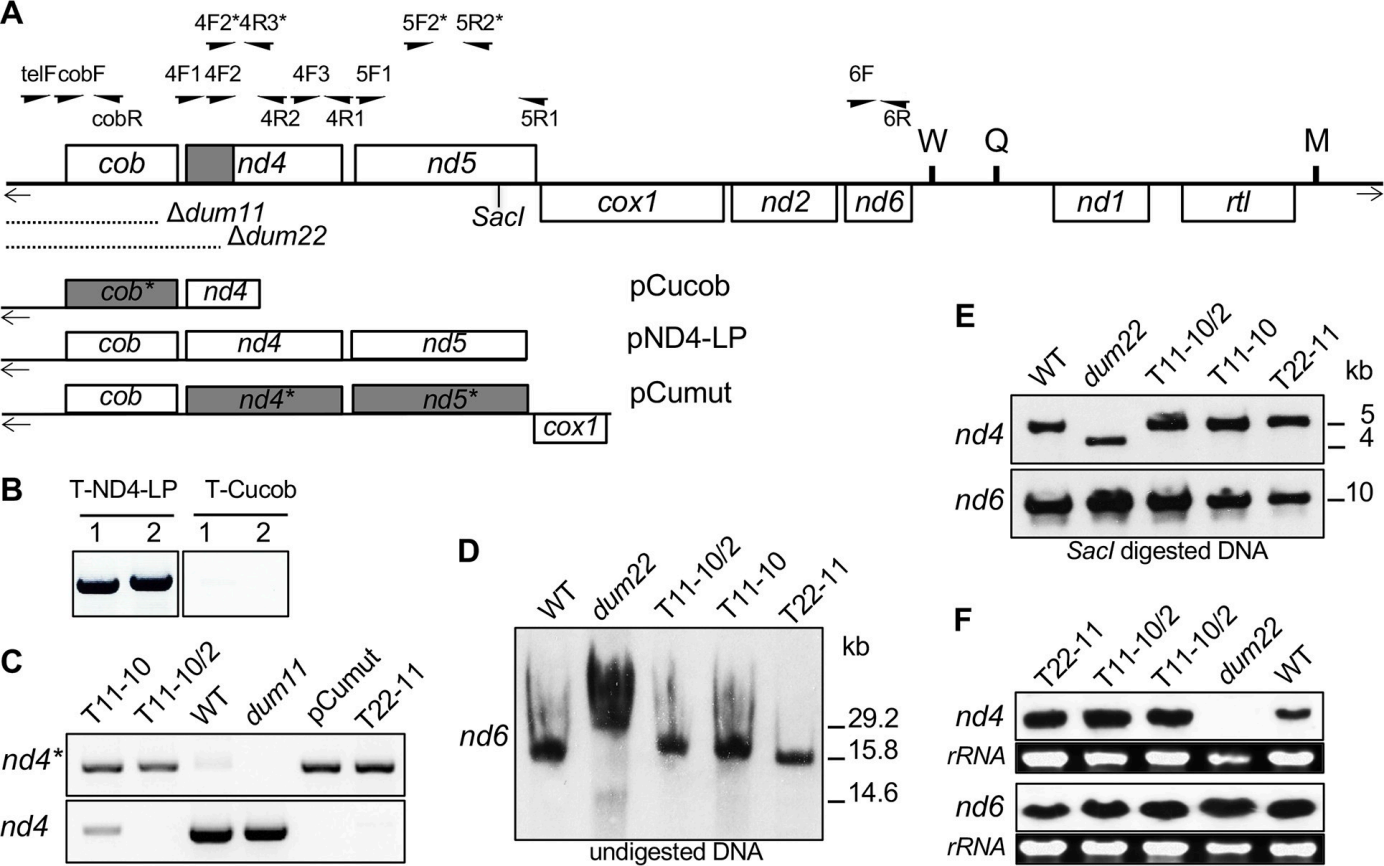
Molecular characterization of the transformants. (A) Schematic map of the *Chlamydomonas reinhardtii* mitochondrial genome. Boxes represent protein-coding genes: (*cob*) apocytochrome *b* of complex III; (*nd1*, *2*, *4*, *5* and *6*) subunits of complex I; (*cox1*) subunit 1 of complex IV; (*rtl*) reverse transcriptase-like protein. W, Q, and M represent tRNAs for Trp, Glu, and Met, respectively. The bidirectional origin of transcription between *nd5* and *cox1* genes is represented by a dashed vertical line and two horizontal arrows. Terminal inverted repeats are shown by short arrows and *Sac*I digestion site at position 5.5 kb (GenBank u03843 numbering) is indicated. Region where modifications on the *nd4* gene were found on T11-10, T11-10/2 and T22-11 transformants is indicated in grey. Position and name of primers are indicated above the map. Primers with a star are specific for the modified gene version (for primer sequence see S1 Table). Positions of the *dum11* and *dum22* deletions are shown. Mitochondrial DNA fragments contained in pND4-LP, pCucob and pCumut are schematized. Grey boxes represent the modified genes where GGC/GGT codons were changed in GGG codons. (B**)** Detection of the *cob* gene in transformants obtained after biolistic transformation with pND4-LP (T-ND4-LP) and pCucob (T-cucob) constructs. PCR analyses were performed with cobF/cobR (1) and telF/cobR (2) pair primers. Gaps between lanes 2 and 3 indicate where lanes from the original image were removed in preparing the figure. Original image data are in S1 File. (C**)** Detection of the mutated and the wild-type *nd4* genes on T11-10, T11-10/2 and T22-11 transformants. PCR analyses were performed with 4F2*/4R2 and 4F2/4R2 pair primers for the modified *nd4* gene (nd4*) and for wild-type *nd4* gene (*nd4*) respectively. (D-E**)** Reconstitution of complete mitochondrial genome in T11-10, T11-10/2 and T22-11 transformants. Southern blot analyses were performed (D) on total DNA with the *nd6* PCR probe and (E) on *Sac*I digested DNA with *nd4* and *nd6* PCR probes. (F**)** Transcript levels of *nd4* and *nd6* genes in T11-10, T11-10/2 and T22-11 transformants. Northern blot analyses were performed on total RNA with *nd4* and *nd6* PCR probes. Loadings of rRNAs are shown.

**Fig 5:**
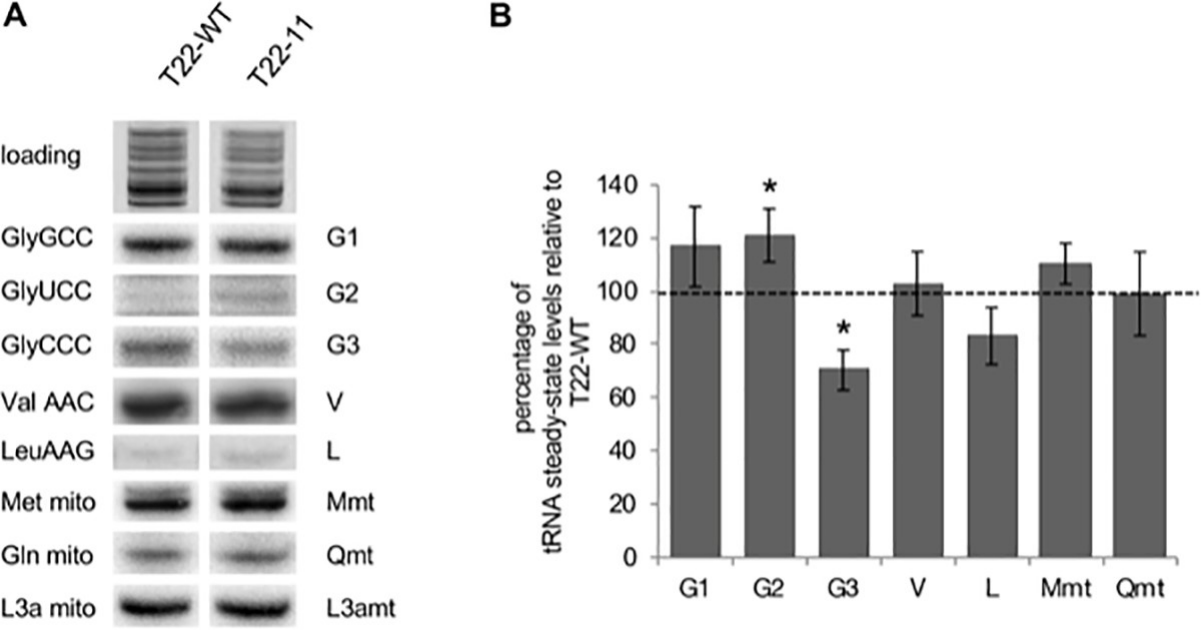
Analysis of the import status of mitochondrial tRNAs in T22-11 transformant. (A) Northern blot analysis of mitochondrial tRNAs extracted from the T22-WT strain and T22-11 transformant. Hybridizations were performed with radiolabeled oligonucleotides specific for cytosolic tRNA^Gly^(GCC) (G1), tRNA^Gly^(UCC) (G2), tRNA^Gly^(CCC) (G3), tRNA^Val^(AAC) (V) and tRNA^Leu^(AAG) (L); for mitochondrial tRNA^Met^ (M mt), tRNA^Gln^ (Q mt) and for the mitochondrial L3a rRNA (L3a mt). Gaps between lanes 1 and 2 indicate where lanes from the original image were removed in preparing the figure; original images are in S1 File. (B) Signals were quantified and normalized with the L3a mt signal. Results are the means of 3 to 5 independent experiments and correspond to the percentage of variation of tRNA steady-state levels in the T22-11 transformant as compared to the T22-WT strain. Asterisks indicate statistically significant differences using Student t test with a significance threshold of 0.05.

## Supporting information

S1 File Original image data supporting Figs 1B, 1D and 2A. Results shown in Fig 2A were obtained using multiple northern blots, one of which was stripped and reprobed multiple times. The corresponding input data for each northern blot are included in S1 File.
(PDF)

S2 File Quantitative data supporting Fig 2B. (PDF)
