# Supplementary material for: Co-Evolution of Mitochondrial tRNA Import and Codon Usage Determines Translational Efficiency in the Green Alga Chlamydomonas
Source: PLoS Genet. 2012 Sep 20;8(9):e1002946. doi: 10.1371/journal.pgen.1002946 (PMC3447967; doi:10.1371/journal.pgen.1002946)
Supplement: Figure S1 — Alignment of the wild-type and the modified genes. Modified nucleic acids are indicated in white. (PDF) [file pgen.1002946.s001.pdf]

Figure S1

```
modified_cob   ATGCGTATGCATAACAAAATTCAATTGTTGAGTGTACTAAACACTCATTTGGTAGCCTACCCAACTCCAATGAACCTAAACTATTCTTGGAACGGGGGGTCTCTAGCTGGGATGA
wild-type_cob  ATGCGTATGCATAACAAAATTCAATTGTTGAGTGTACTAAACACTCATTTGGTAGCCTACCCAACTCCAATGAACCTAAACTATTCTTGGAACGGTGGTTCTCTAGCTGGTATGA

modified_cob   TGCTAGCTAGTCAAATGCTTACTGGGATTCTACTAGCCATGCACTATGTAGGGCACGTAGACTACGCTTTTGCTAGCGTACAACACCTAATGACTGATGTACCTTCTGGGATGAT
wild-type_cob  TGCTAGCTAGTCAAATGCTTACTGGTATTCTACTAGCCATGCACTATGTAGGTCACGTAGACTACGCTTTTGCTAGCGTACAACACCTAATGACTGATGTACCTTCTGGTATGAT

modified_cob   CTTGCGTTACGCTCACGCTAACGGGGCCAGCTTGTTCTTTATTGTAGTCTATTTGCACGTATTGCGTGGGATGTACTACGGGAGCGGGGCTCAGCCACGTGAGATCGTCTGGATC
wild-type_cob  CTTGCGTTACGCTCACGCTAACGGCGCCAGCTTGTTCTTTATTGTAGTCTATTTGCACGTATTGCGTGGTATGTACTACGGTAGCGGCGCTCAGCCACGTGAGATCGTCTGGATC

modified_cob   AGTGGGGTCGTTATCTTGTTGGTAATGATTATCACCGCCTTCATTGGGTATGTACTACCATGGGGGCAAATGTCTTTCTGGGGGGCTACCGTAATTACTAGTTTGGCTACTGCCA
wild-type_cob  AGTGGTGTCGTTATCTTGTTGGTAATGATTATCACCGCCTTCATTGGTTATGTACTACCATGGGGCCAAATGTCTTTCTGGGGTGCTACCGTAATTACTAGTTTGGCTACTGCCA

modified_cob   TTCCAGTAGTAGGGAAACACATCATGTACTGGTTGTGGGGGGGGTTCAGTGTTGATAACCCAACCTTGAACCGCTTCTACAGCTTCCACTACACTCTACCACTCATCTTGGCTGG
wild-type_cob  TTCCAGTAGTAGGTAAACACATCATGTACTGGTTGTGGGGTGGTTTCAGTGTTGATAACCCAACCTTGAACCGCTTCTACAGCTTCCACTACACTCTACCACTCATCTTGGCTGG

modified_cob   GTTGAGCGTATTCCACATTGCCGCCTTGCACCAATACGGGAGTACTAACCCACTAGGGGTTAACAGCCAAAGCAGCCTAATTTCTTTCGGGTCTTACTTTGGGGCTAAAGACCTG
wild-type_cob  TTTGAGCGTATTCCACATTGCCGCCTTGCACCAATACGGTAGTACTAACCCACTAGGTGTTAACAGCCAAAGCAGCCTAATTTCTTTCGGTTCTTACTTTGGTGCTAAAGACCTG

modified_cob   GTCGGGGCTTTGTTCTTGGCTCTTGTGTTCAGCATTCTAGTCTTCTTCTACCCAGACTTGTTGGGGCACCCAGACAACCTAATCCCAGCTAACCCATATAGCACCCCACAACACA
wild-type_cob  GTCGGTGCTTTGTTCTTGGCTCTTGTGTTCAGCATTCTAGTCTTCTTCTACCCAGACTTGTTGGGTCACCCAGACAACCTAATCCCAGCTAACCCATATAGCACCCCACAACACA

modified_cob   TTGTACCAGAGTGGTACTTCTTGTGGGTATACGCTATTCTACGTTCCATTCCAAACAAAGCTATGGGGGTATTGGCTATTGGGCTAGTCTTCGCTAGTTTGTTTGCTATGCCATT
wild-type_cob  TTGTACCAGAGTGGTACTTCTTGTGGGTATACGCTATTCTACGTTCCATTCCAAACAAAGCTATGGGCGTATTGGCTATTGGTCTAGTCTTCGCTAGTTTGTTTGCTATGCCATT

modified_cob   CATCGGGTTGGGGGGGGGGAAATTCCGCATCATCACTGAGTGGCTATACTGGACTTTCCTTGCTGATGTATTGCTATTGACCTGGTTGGGGGGGAACGAGATTACTCCAATTACC
wild-type_cob  CATCGGTTTGGGCGGTGGTAAATTCCGCATCATCACTGAGTGGCTATACTGGACTTTCCTTGCTGATGTATTGCTATTGACCTGGTTGGGTGGTAACGAGATTACTCCAATTACC

modified_cob   TCTTTCGTCGGACAGTGCTGCACTGCTTACCTATTCTTCTACCTACTTGTTTGTCAACCACTTGTAGGGTACTTGGAGACTCAGTTTGCCCACGGGACTCAAACCAACTAA
wild-type_cob  TCTTTCGTCGGACAGTGCTGCACTGCTTACCTATTCTTCTACCTACTTGTTTGTCAACCACTTGTAGGTTACTTGGAGACTCAGTTTGCCCACGGTACTCAAACCAACTAA
```
