# Supplementary figures and images for: Co-Evolution of Mitochondrial tRNA Import and Codon Usage Determines Translational Efficiency in the Green Alga Chlamydomonas
Source: PLoS Genet. 2012 Sep 20;8(9):e1002946. doi: 10.1371/journal.pgen.1002946 (PMC3447967; doi:10.1371/journal.pgen.1002946)

Figure S2

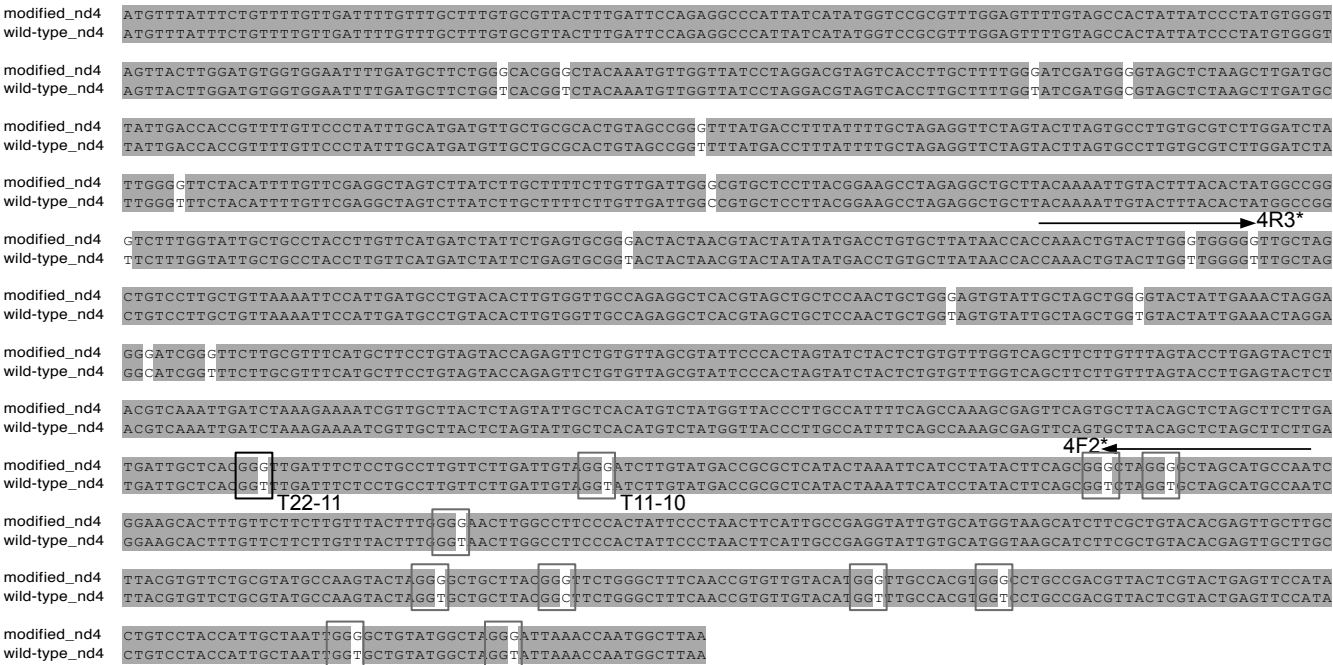

Supplement: Figure S2 — Alignment of the wild-type and the modified nd4 gene. Modified nucleic acids are indicated in white. Position and name of the oligonucleotides specific for modified nd4 gene are indicated by a long arrow. The ten modified GGC/GGT codons into GGG codons in the T11-10 transformant are framed in grey. The additional codon found in the T22-11 transformant is framed in black. (PDF) [file pgen.1002946.s002.pdf]
