# Supplementary material for: Co-Evolution of Mitochondrial tRNA Import and Codon Usage Determines Translational Efficiency in the Green Alga Chlamydomonas
Source: PLoS Genet. 2012 Sep 20;8(9):e1002946. doi: 10.1371/journal.pgen.1002946 (PMC3447967; doi:10.1371/journal.pgen.1002946)
Supplement: Figure S3 — Alignment of the wild-type and the modified nd5 gene. Modified nucleic acids are indicated in white. Position and name of oligonucleotides specific for modified nd5 gene are indicated by a long arrow. (PDF) [file pgen.1002946.s003.pdf]

Figure S3

```
modified_nd5   ATGTTTTTGTTGTCTGTTTTCTTTCCCCTACTAGGGGGGCTTGTTAACACTTCCCCAATTGCTCGTTTTCTAGGGCATCGTGGGAGTAGTATTATTGCTATTGGGTGCATGGT
wild-type_nd5  ATGTTTTTGTTGTCTGTTTTCTTTCCCCTACTAGGTGGTCTTGTTAACACTTCCCCAATTGCTCGTTTTCTAGGTCATCGTGGTAGTAGTATTATTGCTATTGGCTGCATGGT

modified_nd5   GGTAGCCTTTATCTCTAGCGTTGTAATCTATTACGAGGTTGTATTTATGGGGTGCGCGGTAAGCGTCGACGTCTTTGGGACTTGGTTCTCCGTCGGGACCTTTCATGCCGGGT
wild-type_nd5  GGTAGCCTTTATCTCTAGCGTTGTAATCTATTACGAGGTTGTATTTATGGGCTGCGCGGTAAGCGTCGACGTCTTTGGTACTTGGTTCTCCGTCGGTACCTTTCATGCCGGTT

modified_nd5   GGACTTTTAACTTCGACCTACTAACCGCCAACATGCTATTCACCGTTACCGGGGTAAGTATGGCCGTACACATGTACGCCTGTGACTACATGCGTCAAGATCCCCACTTGAAC
wild-type_nd5  GGACTTTTAACTTCGACCTACTAACCGCCAACATGCTATTCACCGTTACCGGTGTAAGTATGGCCGTACACATGTACGCCTGTGACTACATGCGTCAAGATCCCCACTTGAAC
                                                                                                                   5R2*

modified_nd5   TTGTTTTTGGGGTATCTAAGCTACTTTACTGGGTTTATGTGCGTACTTGTAGCCGCTGATAACTTGTTGGTTATGCTTGTTGGGTGGGAGGGGATCGGGGTTTGTTCCTACCT
wild-type_nd5  TTGTTTTTGGGTTATCTAAGCTACTTTACTGGTTTTATGTGCGTACTTGTAGCCGCTGATAACTTGTTGGTTATGCTTGTTGGTTGGGAGGGTATCGGTGTTTGTTCCTACCT

modified_nd5   ATTGATCGGGTACTGGAGTCATCGTCTATCCGCCGTTAAAAGTGCTCAAAAAGCCATTTTGGTTAACCGTGTTAGTGATGGGTTGCTAATGTGGGGGGTCTTGTGGGTATGGT
wild-type_nd5  ATTGATCGGTTACTGGAGTCATCGTCTATCCGCCGTTAAAAGTGCTCAAAAAGCCATTTTGGTTAACCGTGTTAGTGATGGTTTGCTAATGTGGGGTGTCTTGTGGGTATGGT

modified_nd5   ACCATTTGGGGAGTCTAGAGTATGATTTGCTTAACGTTTATTCTGCCAGCGGGTTTGTAGGGCTTTCTATCTTGATTGGGGCTATGGGGAAATCCGCTCAGATTTTGTTCCAC
wild-type_nd5  ACCATTTGGGTAGTCTAGAGTATGATTTGCTTAACGTTTATTCTGCCAGCGGCTTTGTAGGCCTTTCTATCTTGATTGGTGCTATGGGTAAATCCGCTCAGATTTTGTTCCAC

modified_nd5   GTTTGGCTTGCTGATGCCATGGAGGGGCCAACCCCAGTATCCGCCCTAATTCACGCCGCTACTCTGGTCACTGCTGGGGTATACTTGCTTGTACGCTTGCATATCCACGATGA
wild-type_nd5  GTTTGGCTTGCTGATGCCATGGAGGGCCCAACCCCAGTATCCGCCCTAATTCACGCCGCTACTCTGGTCACTGCTGGTGTATACTTGCTTGTACGCTTGCATATCCACGATGA

modified_nd5   GATGTTTGTTATCATCGTAGGGAGCTTGACCGCTTTTATGGCTGGGGTGTTCGGGGCCACTCAAAGTGATTTGAAACGTGTCATTGCTTACAGTACTTGCAGTCAACTTGGGT
wild-type_nd5  GATGTTTGTTATCATCGTAGGTAGCTTGACCGCTTTTATGGCTGGTGTGTTCGGTGCCACTCAAAGTGATTTGAAACGTGTCATTGCTTACAGTACTTGCAGTCAACTTGGCT

modified_nd5   ATATGATGGTTAGTCTGGGGCTAGGGGAGACTGGGGGGAGGCTAGCATGGGGCACCTTATGACTCACGCTAGTTTTAAAGCCGCTCTATTCTTGGCCGCCGGGATGGTTATT
wild-type_nd5  ATATGATGGTTAGTCTGGGCCTAGGCGAGACTGGCGGTGAGGCTAGCATGGGTCACCTTATGACTCACGCTAGTTTTAAAGCCGCTCTATTCTTGGCCGCCGGTATGGTTATT
                5F2*

modified_nd5   AGCGGGAACGGGGGGAACCAGCACATCGCTCGTTACGGGGGGAGTGCTCACAGTGCTATGTTCACTATGCTAACCTTGATGGTCGCTTCCTTGAGTTTGATTGGGTGGCCAGA
wild-type_nd5  AGCGGTAACGGTGGTAACCAGCACATCGCTCGTTACGGTGGTAGTGCTCACAGTGCTATGTTCACTATGCTAACCTTGATGGTCGCTTCCTTGAGTTTGATTGGCTGGCCAGA

modified_nd5   GCTAAGTGGGTTCTATTCTAAAGAGACCATCTTGAACTTGGCCGCTATTTGCGCTGATCCAATTGCCGATGTCGCTCATACTTTGCTATTGCTAACTGCTATGCTAACCAGTG
wild-type_nd5  GCTAAGTGGTTTCTATTCTAAAGAGACCATCTTGAACTTGGCCGCTATTTGCGCTGATCCAATTGCCGATGTCGCTCATACTTTGCTATTGCTAACTGCTATGCTAACCAGTG

modified_nd5   CTTACACCACTAAGTTGTTCTATCAGTGCTTTATGGTTGATTTCTCTGGGTCTAGTGTAACCCCAGTTCGCAATGTTTTGCCAATTTTGGCTATGGCTATTTTGTTGCTAGAC
wild-type_nd5  CTTACACCACTAAGTTGTTCTATCAGTGCTTTATGGTTGATTTCTCTGGTTCTAGTGTAACCCCAGTTCGCAATGTTTTGCCAATTTTGGCTATGGCTATTTTGTTGCTAGAC

modified_nd5   ATTATGCTAAAAGTATGGGTAGGGACCAACTTGTTGTCCGGGATGTTGTTCTTCCTACCATGGGGGGTTAAAACTCTACCTTTTGGGTTGATGGTTGCCGGGATTTTGACTGC
wild-type_nd5  ATTATGCTAAAAGTATGGGTAGGTACCAACTTGTTGTCCGGTATGTTGTTCTTCCTACCATGGGGCGTTAAAACTCTACCTTTTGGTTTGATGGTTGCCGGCATTTTGACTGC

modified_nd5   CACTGCTGCTGTTGGGTCTGAGCGTTTTACTTTGATTCGTTTCTGTGGGTCTCGCTGGGGGTTCGATCAATTGTTCGCTCGCAGTCCAGTAAACCCAATCTTTGACCTAGGGC
wild-type_nd5  CACTGCTGCTGTTGGTTCTGAGCGTTTTACTTTGATTCGTTTCTGTGGCTCTCGCTGGGGTTTCGATCAATTGTTCGCTCGCAGTCCAGTAAACCCAATCTTTGACCTAGGTC

modified_nd5   GAATCACTTGGGCTATTGGGGACCGCGGGTTGCTAAGCGTTGGGAATTTGCGTGCTTAA
wild-type_nd5  GAATCACTTGGGCTATTGGTGACCGCGGTTTGCTAAGCGTTGGTAATTTGCGTGCTTAA
```
