# Supplementary material for: Co-Evolution of Mitochondrial tRNA Import and Codon Usage Determines Translational Efficiency in the Green Alga Chlamydomonas
Source: PLoS Genet. 2012 Sep 20;8(9):e1002946. doi: 10.1371/journal.pgen.1002946 (PMC3447967; doi:10.1371/journal.pgen.1002946)
Supplement: Table S1 — Oligonucleotides used for molecular characterization of the transformants. Position indicates the location of the oligonucleotide in the Chlamydomonas mitochondrial genome according to the GenBank u03843 numbering. (PDF) [file pgen.1002946.s004.pdf]

Table S1

Name	Position	Oligonucleotide sequence
**telF**	520-538	GTAAAAAGTGTGTCAATGC
**cobF**	595-614	GTGGTTGACAAACAAGTAGG
**cobR**	1165-1144	TTGAACCGCTTCTACAGCTTCC
**4F1**	1692-1711	GTAAATAATAAATAACAAAG
**4F2**	2003-2022	TTGGCATGCTAGCACCTAGA
**4F2***	2003-2022	TTGGCATGCTAGCCCCTAGC
**4F3**	2765–2780	CAGTGCGCAGCAACAT
**4R3***	2492-2073	CAAACTGTACTTGGTTGGGGT
**4R1**	3091-3071	CTCAATAGTGGTGCTCGTCAC
**4R2**	2542-2523	CTGAGTGCGGTACTACTAAC
**5F1**	3247-3265	CATTGAAACAAGCACGCAG
**5F2***	3920-3938	GTGCTGGTTCCCCCCGTTC
**5R1**	4952-4933	GTTGTCTGTTTTCTTTCCCC
**5R2***	4430-4523	GGCTAGGGGAGACTGGGGGG
**6F**	7996-8015	GCATTGCCGTAGGATACACC
**6R**	8409-8391	CGAAACGACCAAACAGAGC
