# Supplementary material for: Co-Evolution of Mitochondrial tRNA Import and Codon Usage Determines Translational Efficiency in the Green Alga Chlamydomonas
Source: PLoS Genet. 2012 Sep 20;8(9):e1002946. doi: 10.1371/journal.pgen.1002946 (PMC3447967; doi:10.1371/journal.pgen.1002946)
Supplement: Table S2 — Oligonucleotides used for Northern analysis on mitochondrial tRNA fractions. (PDF) [file pgen.1002946.s005.pdf]

Table S2

Name	tRNA target	tRNA sequence and the oligonucleotide position underlined
**G1**	Gly GCC	GCACTAGTGGTATAGTGGTAGCATAGGTCCTT**GCC**ACGGACCAGACCCGAGTTCGATTCTCGGCTAGTGCA
**G2**	Gly UCC	GCGCTACTAGTCCAGTGGTTAGGATATTTGCCT**TCC**AAGCAAAAGAGCCGGGTTCGATTCCCGGGTAGGGCA
**G3**	Gly CCC	GCGCTAGTGGTGTAGTAGCAGCATTTACGCCT**CCC**ACGCGTAAGACTCGGGTGCAAATCCCGGCTAGCGCA
**Mm**	Met CAU mt	AGACACGTAGCTTAGTGGTAAAGCACTGGTCT**CAT**AAGCCAGCTATCGCAAGTTCGAATCTCGCCGTGTCTA
**Qm**	Gln TTG mt	TGGGGCATAGCCAAGTGGTAAGGCATTGGACT**TTG**ACTCCAAGATGCATGGGTTCGAATCCTATTGCCCCAG
**L**	Leu AAG	GGCGATGTGGCCGAGTGGTCtAAGGCGCAGGATT**AAG**GCTCCTGTCCGCAAGGGCGTAGGTTCGAACCCTACCATCGTCA
**V**	Val AAC	GGAATCATGGTGTAGTTGGTTATCACACCTGTCT**AAC**ACACAGGAGGTCTCCAGTTCGATCCTGGATGATTCCA
**L3am**	rRNA mt	ATGAGTGCCTAAGCACTCAATTTCGGTGCTCGGTGAAACCGAGCATCCAATACTAAAGAAACTTTACTGGCTTAGTACTGGGACCCCATTTTTAGTATACCTACATTATATACA
