# Supplementary material for: Correction: Co-Evolution of Mitochondrial tRNA Import and Codon Usage Determines Translational Efficiency in the Green Alga Chlamydomonas
Source: PLoS Genet. 2020 Mar 31;16(3):e1008719. doi: 10.1371/journal.pgen.1008719 (PMC7108720; doi:10.1371/journal.pgen.1008719)
Supplement: S1 File — Results shown in Fig 2A were obtained using multiple northern blots, one of which was stripped and reprobed multiple times. The corresponding input data for each northern blot are included in S1 File. (PDF) [file pgen.1008719.s001.pdf]

**S1 File**. Original image data supporting Figures 1B, 1D and 5A

## Figure 1B

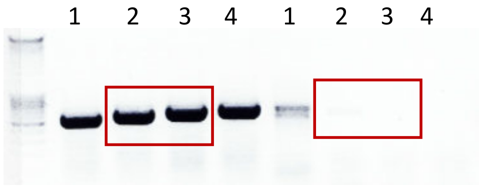


## Figure 1D

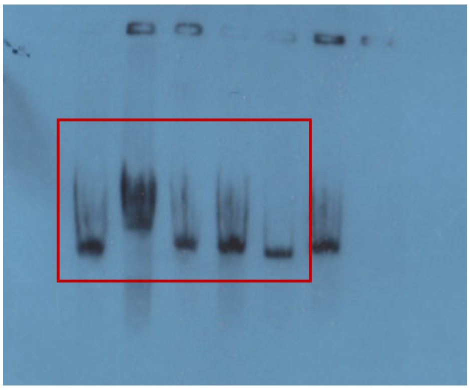

# Figure 5A

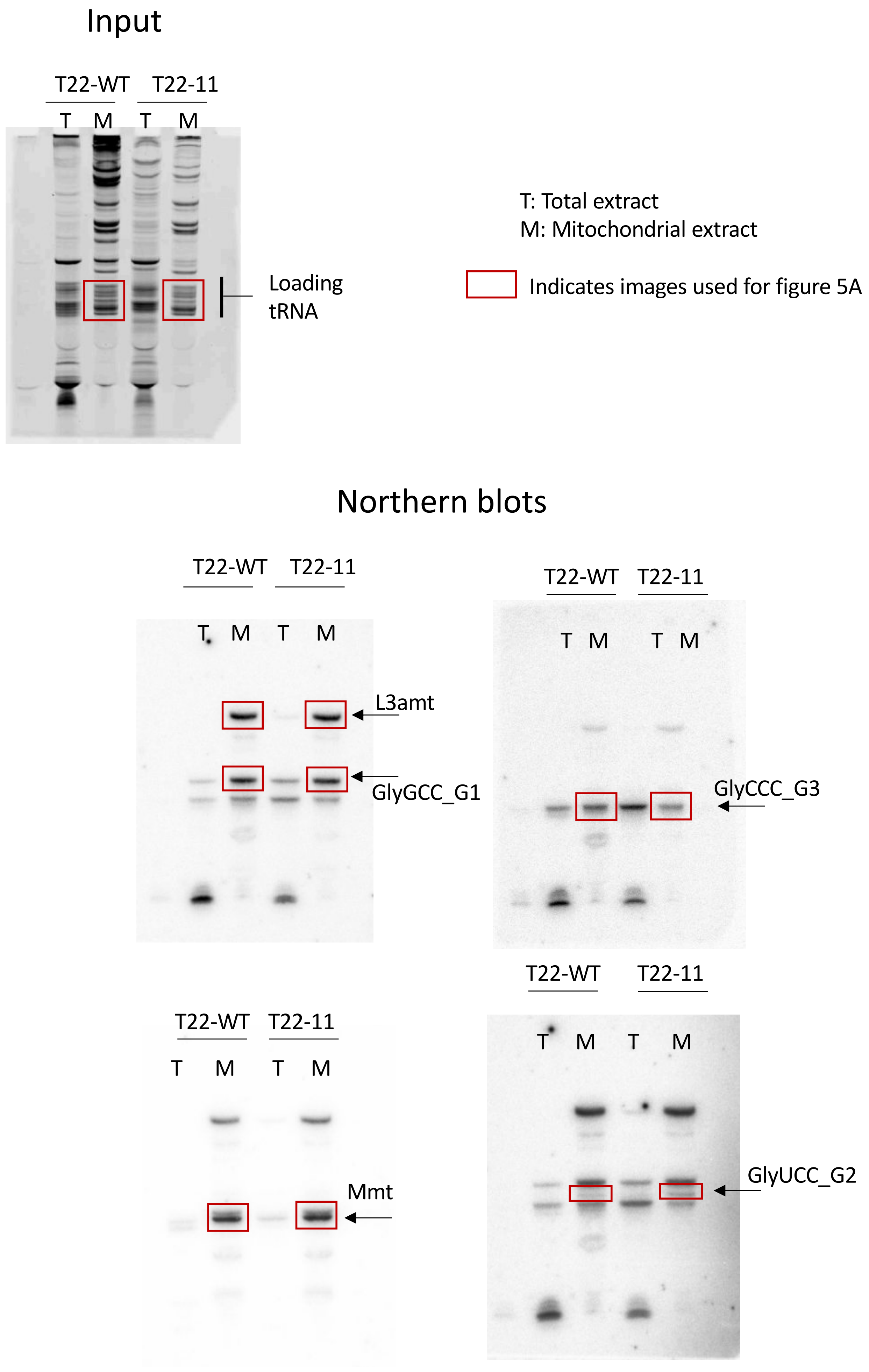
Input
T22-WT
T22-11
T
M
T
M
Loading
tRNA
T: Total extract
M: Mitochondrial extract
Indicates images used for figure 5A
Northern blots
T22-WT
T22-11
T
M
T
M
L3amt
GlyGCC_G1
T22-WT
T22-11
T
M
T
M
GlyCCC_G3
T22-WT
T22-11
T
M
T
M
Mmt
T22-WT
T22-11
T
M
T
M
GlyUCC_G2

**Figure 5A**

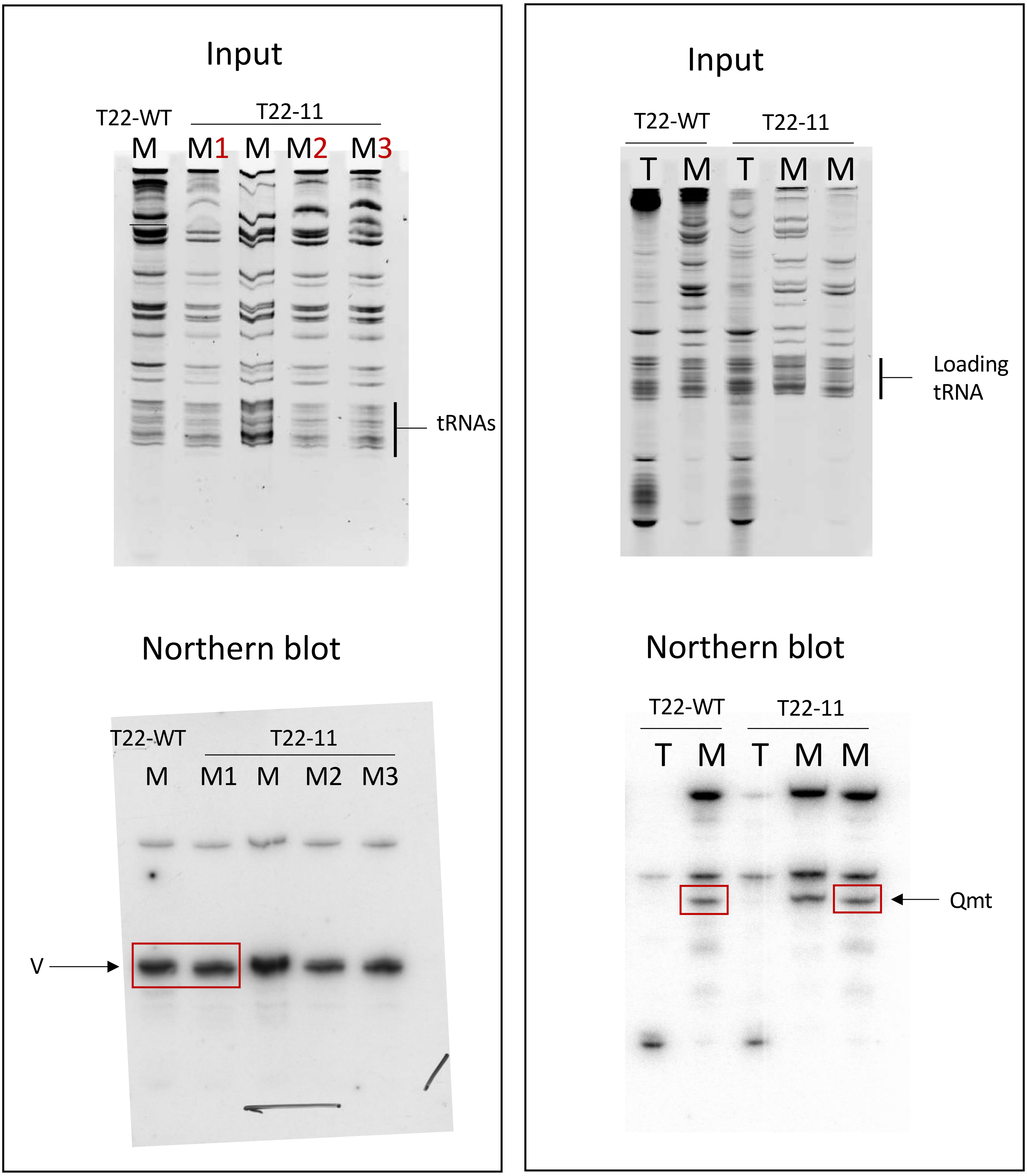
Input
T22-WT
T22-11
M M1 M M2 M3
tRNAs
Northern blot
T22-WT
T22-11
M M1 M M2 M3
V
Input
T22-WT
T22-11
T M T M M
Loading tRNA
Northern blot
T22-WT
T22-11
T M T M M
Qmt

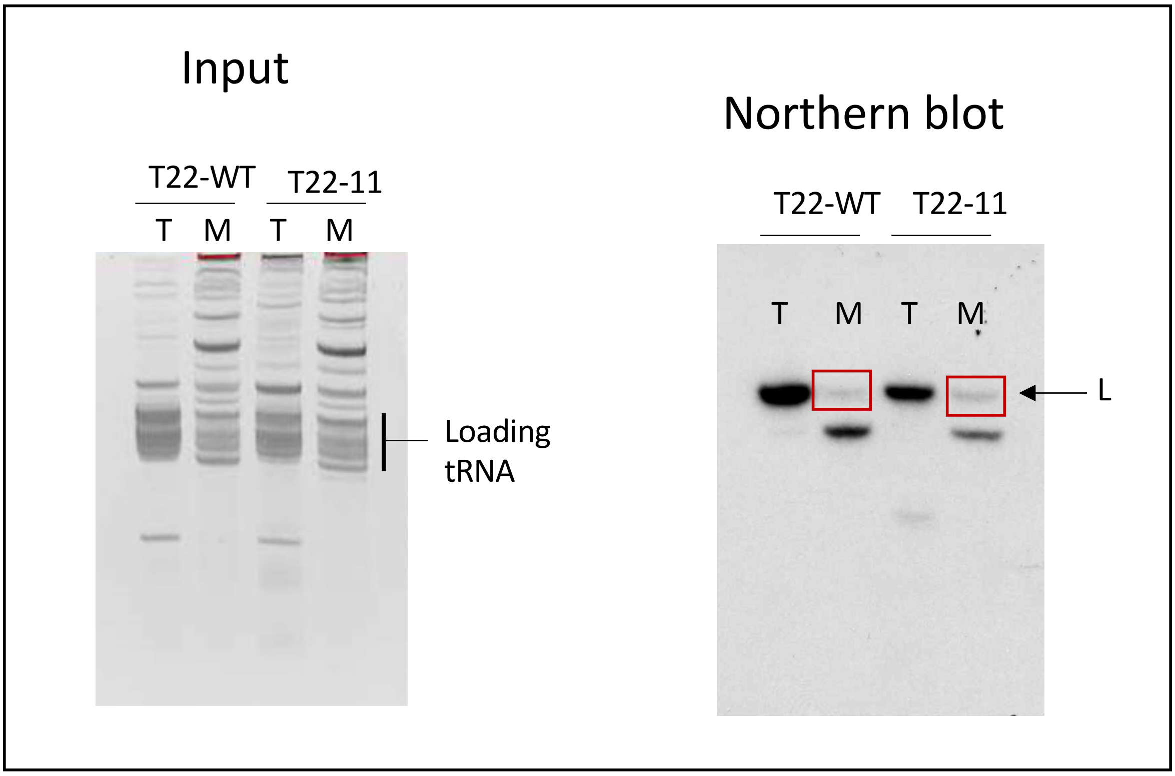
Input
T22-WT
T22-11
T M T M
Loading
tRNA
Northern blot
T22-WT
T22-11
T M T M
L
