# Supplementary material for: Correction: Co-Evolution of Mitochondrial tRNA Import and Codon Usage Determines Translational Efficiency in the Green Alga Chlamydomonas
Source: PLoS Genet. 2020 Mar 31;16(3):e1008719. doi: 10.1371/journal.pgen.1008719 (PMC7108720; doi:10.1371/journal.pgen.1008719)
Supplement: S2 File — (PDF) [file pgen.1008719.s002.pdf]

S2 File: Quantitative data supporting Figure 5B.

Probe_61-Bl.020911

Bl.2.9.11 sd61+1050

NO.	Index	PSL
MtT22-WT	U1	330,3
MtT22-11	U2	317,58
MtT22-WT	U3	2892,7
MtT22-11	U4	1729,8

	61	1050	sd/1050		Log2	%
MtT22-WT	2892,7	330,3	8,758	1,000	0,000	100,0
MtT22-11	1729,8	317,58	5,447	0,622	-0,685	62,2

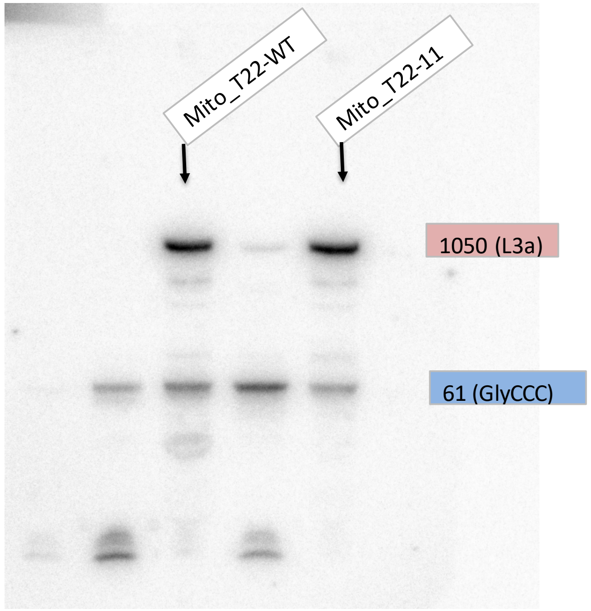

Probe_63-Bl.020911

NO.	Index	PSL	
MtT22-WT	U1	1182,3	1050
MtT22-11	U2	1168,4	
MtT22-WT	U3	218,2	63
MtT22-11	U4	286	
MtT22-WT	U5	647,4	62
MtT22-11	U6	660,6	
			61

Bl.2-09-11_sd63-2.TIFF

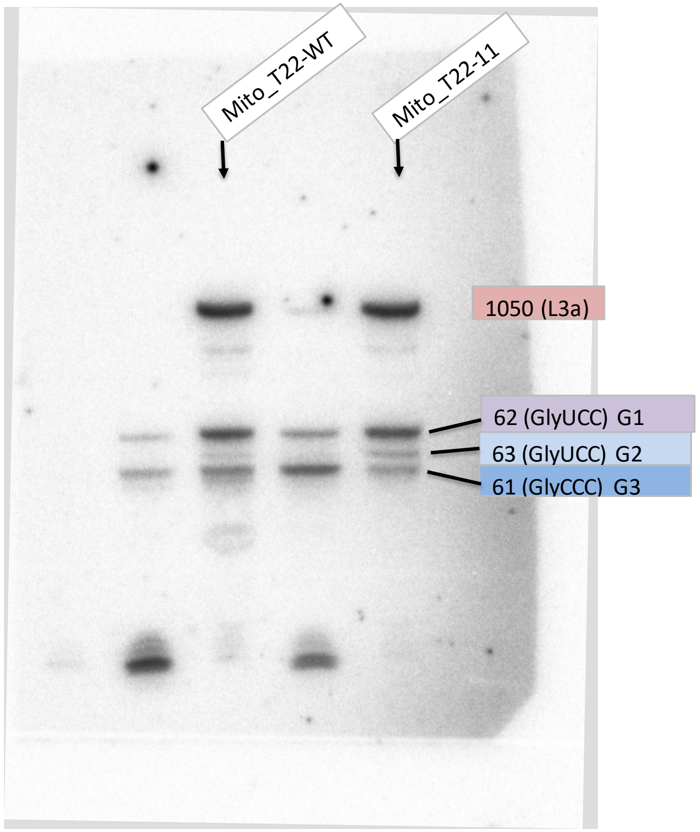

	63	1050	sd/1050		Log2	%
MtT22-WT	218,2	1182,3	0,185	1,000	0,000	100,0
MtT22-11	286	1168,4	0,245	1,326	0,407	132,6

	62	1050	sd/1050		Log2	%
MtT22-WT	647,4	1182,3	0,548	1,000	0,000	100,0
MtT22-11	660,6	1168,3	0,565	1,033	0,046	103,3

Probe_62-61-Bl.020911

NO.	Index	PSL		
MtT22-WT	U1	1738,1	1050	
MtT22-11	U2	1762,5		
MtT22-WT	U3	1434,1	62	Bl.2.9.11 sd62-61
MtT22-11	U4	1427		
MtT22-WT	U5	729	61	
MtT22-11	U6	588		

	61	1050	sd/1050		Log2	%
MtT22-WT	729	1738,1	0,419	1,000	0,000	100,0
MtT22-11	588	1762,5	0,334	0,795	-0,330	79,5

	62	1050	sd/1050		Log2	%
MtT22-WT	1434,1	1738,1	0,825	1,000	0,000	100,0
MtT22-11	1427	1762,5	0,810	0,981	-0,027	98,1

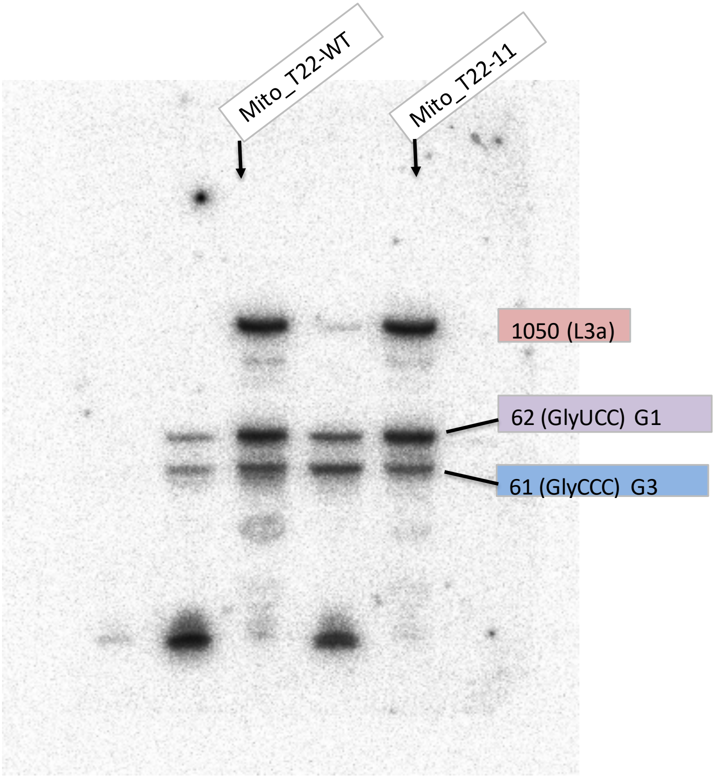

Probe_832-831-Bl.020911

NO.	Index	PSL		
MtT22-WT	U1	1622,2	1050	
MtT22-11	U2	1830,4		
MtT22-WT	U3	3602,6	831	Bl.2.9.11sd-831-832
MtT22-11	U4	3883		
MtT22-WT	U5	1788	832	
MtT22-11	U6	2045,2		

	831	1050	sd/1050		Log2	%
MtT22-WT	3602,6	1622,2	2,221	1,000	0,000	100,0
MtT22-11	3883	1830,4	2,121	0,955	-0,066	95,5

	832	1050	sd/1050		Log2	%
MtT22-WT	1788	1622,2	1,102	1,000	0,000	100,0
MtT22-11	2045,2	1830,4	1,117	1,014	0,020	101,4

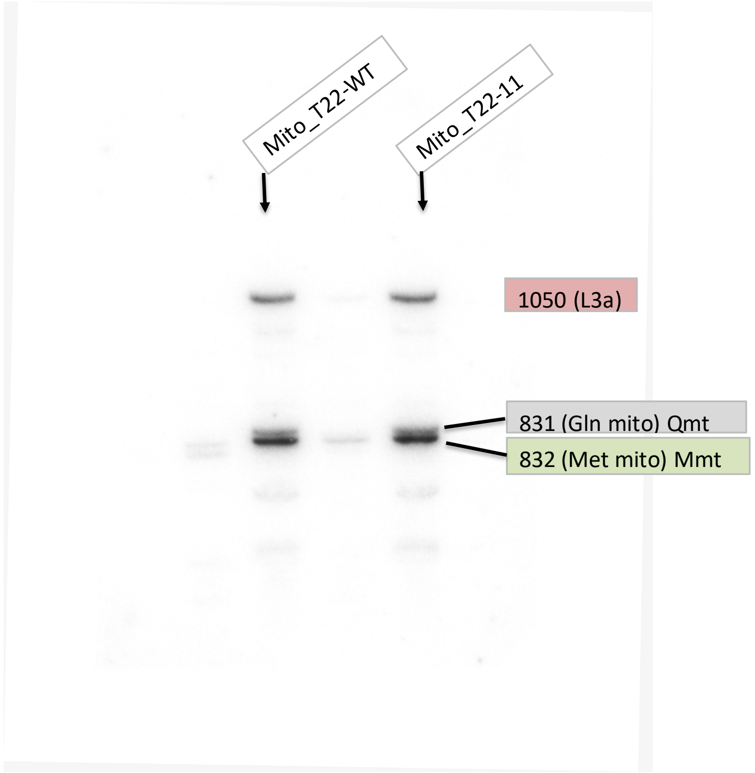

Probe_884-Bl.020911

NO.	Index	PSL				
MtT22-WT	U1	1484	1050			
MtT22-11	U2	1605				
MtT22-WT	U3	13522,7	884			Bl.2-09-11_sd884
MtT22-11	U4	13495,5				

	884 (2)	1050	sd/1050		Log2	%
MtT22-WT	13522,7	1484	9,112	1,000	0,000	100,0
MtT22-11	13495,5	1605	8,408	0,923	-0,116	92,3

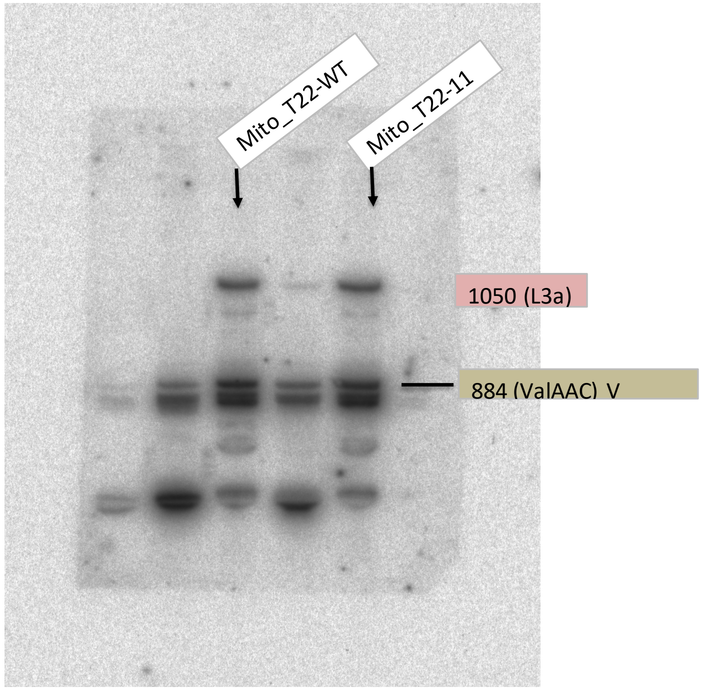

Probe_958-Bl.020911

NO.	Index	PSL	
MtT22-WT	U1	393,28	1050
MtT22-11	U2	424,94	
MtT22-WT	U3	373,47	958
MtT22-11	U4	308,63	

	958	1050	sd/1050		Log2	%
MtT22-WT	373,47	393,28	0,950	1,000	0,000	100,0
MtT22-11	308,63	424,94	0,726	0,765	-0,387	76,5

phosphoimager with probe 958 LeuAAG not found and the scan is provided

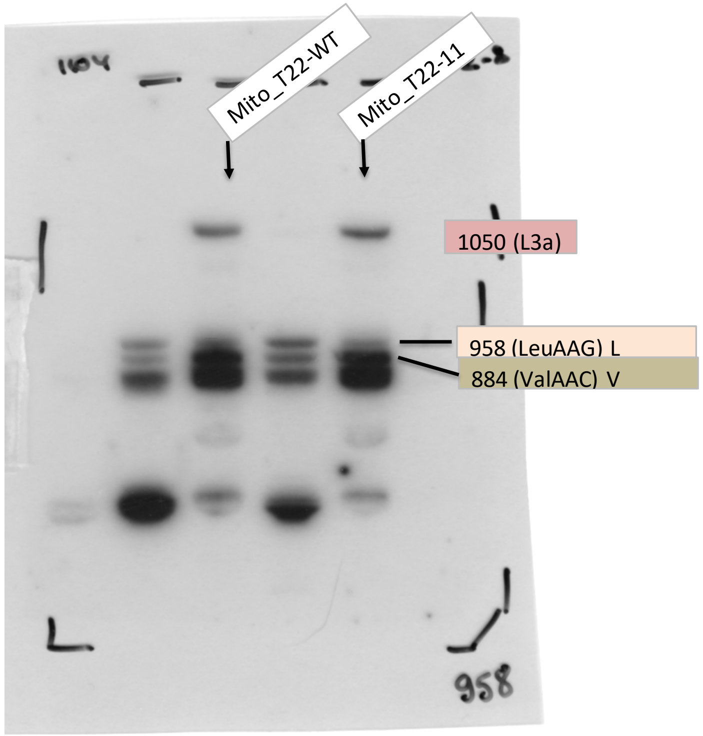

Results-Bl.020911

MtT22-11	61	63	832-831	62-61	884	958			AV	SD
61	62,2								62,2	
62		103,3		98,1					100,7	3,6
63		132,6							132,6	
884					92,3				92,3	
958						76,5			76,5	
832			101,4						101,4	
831			95,5						95,5	

Probe_831-832-Bl.080911

NO.	Index	PSL	
MtT22-WT	U1	57,4	1050
MtT22-11-1	U2	50,6	
MtT22-11-3	U4	61,8	
MtT22-WT	U5	1483,2	832
MtT22-11-1	U6	1634,7	
MtT22-11-3	U8	1837,5	
MtT22-WT	U9	2635,2	831
MtT22-11-1	U10	2498,4	
MtT22-11-3	U12	2419,6	

Bl.8.9.11sd831-2

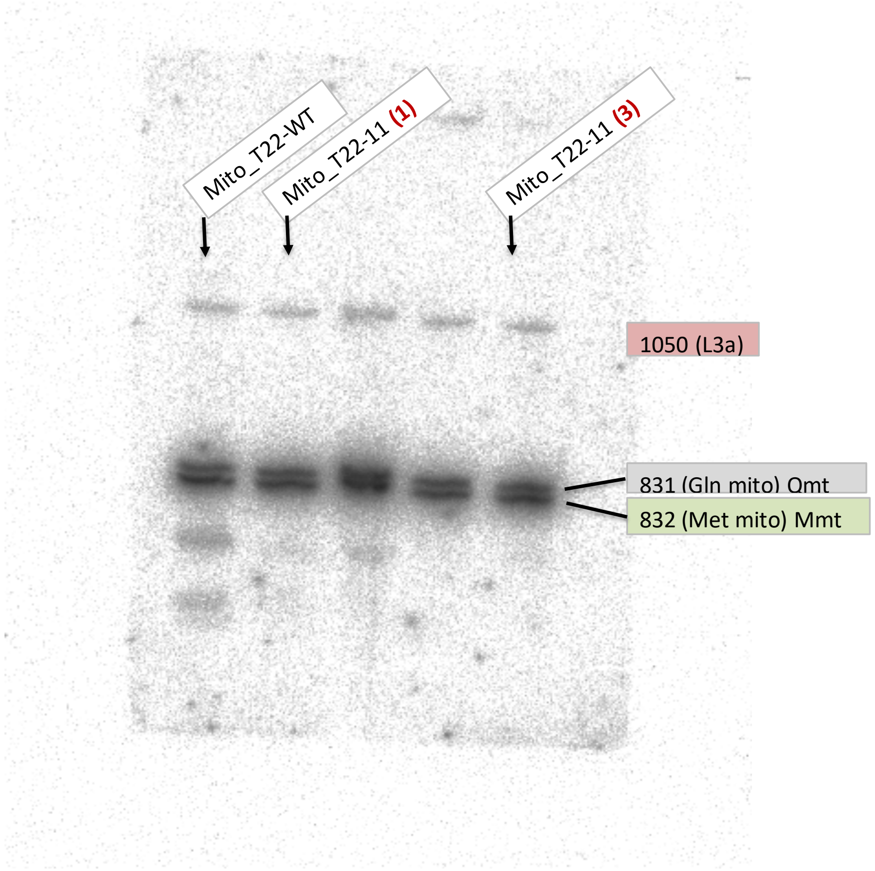

	832	1050	sd/1050		Log2	%
MtT22-WT	1483,2	57,4	25,840	1,000	0,000	100,0
MtT22-11-1	1634,7	50,6	32,306	1,250	0,322	125,0
MtT22-11-3	1837,5	61,8	29,733	1,151	0,202	115,1

	831	1050	sd/1050		Log2	%
MtT22-WT	2635,2	57,4	45,909	1,000	0,000	100,0
MtT22-11-1	2498,4	50,6	49,375	1,075	0,105	107,5
MtT22-11-3	2419,6	61,8	39,152	0,853	-0,230	85,3

Probe_884-Bl.080911

NO.	Index	PSL	
MtT22-WT	U1	222,36	1150
MtT22-11-1	U2	177,68	
MtT22-11-3	U4	224,11	
MtT22-WT	U5	2000,7	884
MtT22-11-1	U6	1848,04	
MtT22-11-3	U8	1871,8	

	884	1050	sd/1050		Log2	%
MtT22-WT	2000,7	222,36	8,998	1,000	0,000	100,0
MtT22-11-1	1848,04	177,68	10,401	1,156	0,209	115,6
MtT22-11-3	1871,8	224,11	8,352	0,928	-0,107	92,8

phosphoimager not found and the scan is provided

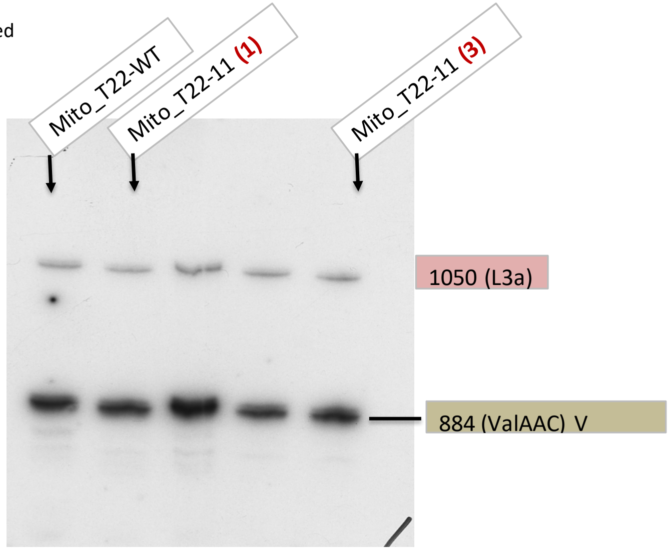

Probe_958-Bl.080911

NO.	Index	PSL	
MtT22-WT	U1	234,32	1150
MtT22-11-1	U2	194,37	
MtT22-11-3	U4	227,95	
MtT22-WT	U5	126,9	958
MtT22-11-1	U6	100,47	
MtT22-11-3	U8	95,69	

	958	1050	sd/1050		Log2	%
MtT22-WT	126,9	234,32	0,542	1,000	0,000	100,0
MtT22-11-1	100,47	194,37	0,517	0,954	-0,067	95,4
MtT22-11-3	95,69	227,95	0,420	0,775	-0,367	77,5

Results-Bl.080911

MtT22-11	series_1	series_3	AV	SD
831	107,5	85,3	96,4	15,7
832	125,0	115,1	120,0	7,0
884	115,6	71,5	93,6	31,2
958	95,4	70,5	83,0	17,6

Probe_62-Bl.210611

NO.	Index	PSL
MtT22-WT	U2	4647,37
MtT22-11	U3	5698,78
MtT22-WT	U8	9442,08
MtT22-11	U9	8848,49

	62	1050	sd/1050		Log2	%
MtT22-WT	4647,37	9442,08	0,492	1,000	0,000	100,0
MtT22-11	5698,78	8848,49	0,644	1,308	0,388	130,8

sd62 bl.21.6.11

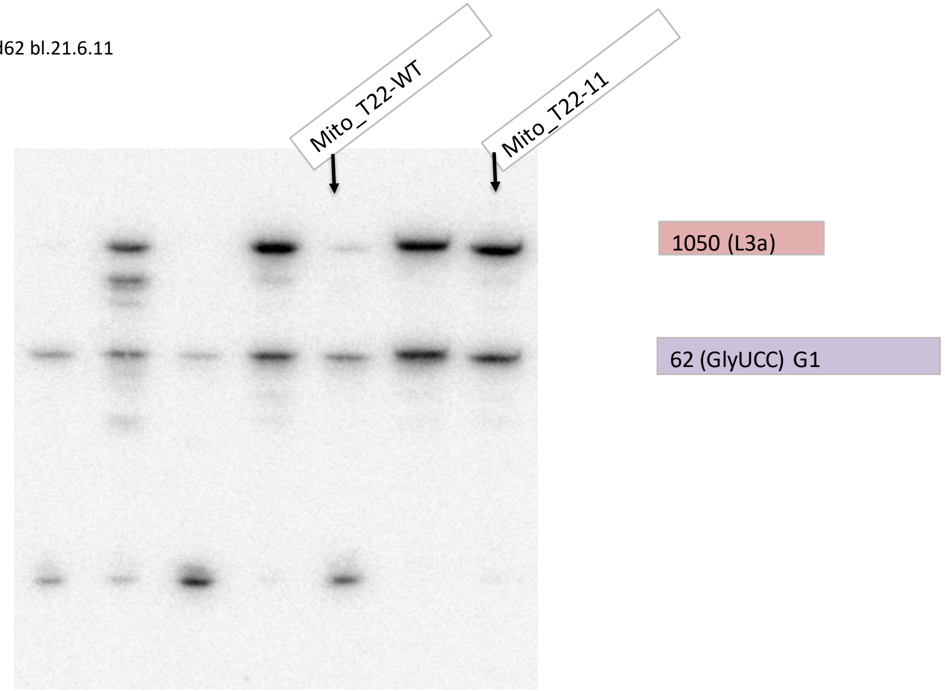

Probe_831-Bl.210611

NO.	Index	PSL
MtT22-WT	U2	1534,54
MtT22-11	U3	1742,81
MtT22-WT	U8	6539,88
MtT22-11	U9	6395,12
MtT22-WT	U14	2224,02
MtT22-11	U15	2752,26

	62	1050	sd/1050		Log2	%
MtT22-WT	2224,02	6539,88	0,340	1,000	0,000	100,0
MtT22-11	2752,26	6395,12	0,430	1,266	0,340	126,6

	831	1050	sd/1050		Log2	%
MtT22-WT	1534,54	6539,88	0,235	1,000	0,000	100,0
MtT22-11	1742,81	6395,12	0,273	1,161	0,216	116,1

sd831 bl.21.6.11

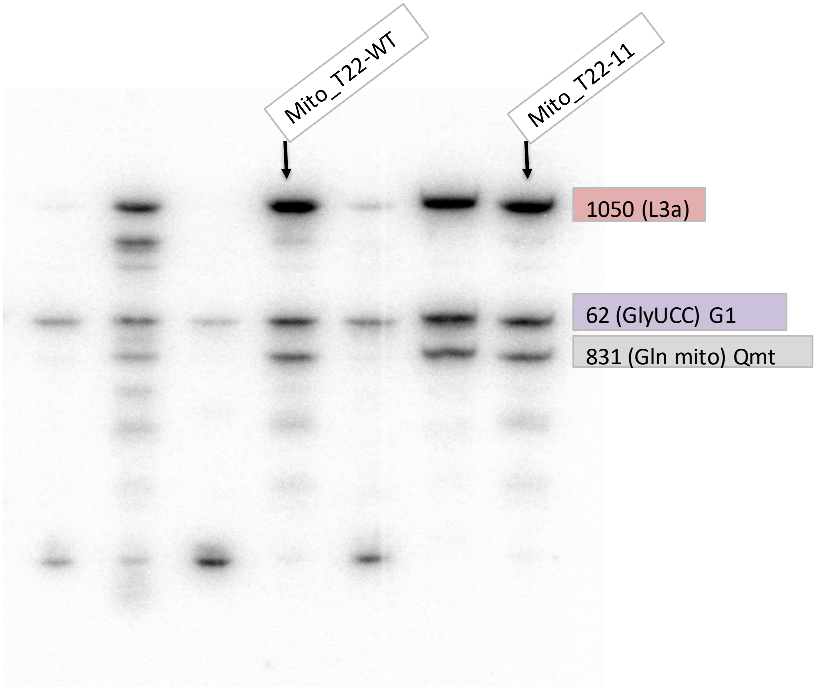

Probe_61-Bl.210611

NO.	Index	PSL
MtT22-WT	U1	445,4
MtT22-11	U2	376
MtT22-WT	U3	1853,5
MtT22-11	U4	1125

Bl. 21.6.11 sd61

	61	1050	sd/1050		Log2	%
MtT22-WT	1853,5	445,4	4,161	1,000	0,000	100,0
MtT22-11	1125	376	2,992	0,719	-0,476	71,9

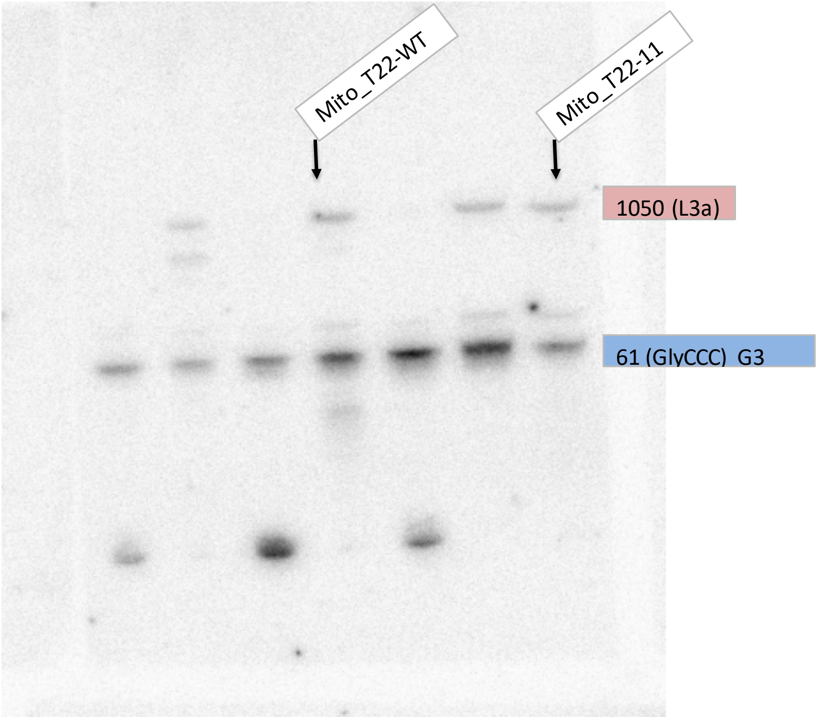

Probe_63-Bl.210611

NO.	Index	PSL		
MtT22-WT	U1	420,5	1050	
MtT22-11	U2	356,6		
MtT22-WT	U3	432,2	63	Bl.21.6.11 sd63
MtT22-11	U4	424,1		
MtT22-WT	U5	864,5	62?	
MtT22-11	U6	1107,8		
MtT22-WT	U7	1230	61	
MtT22-11	U8	730,5		

	63	1050	sd/1050		Log2	%
MtT22-WT	432,2	420,5	1,028	1,000	0,000	100,0
MtT22-11	424,1	356,6	1,189	1,157	0,211	115,7

	62	1050	sd/1050		Log2	%
MtT22-WT	864,5	420,5	2,056	1,000	0,000	100,0
MtT22-11	1107,8	356,6	3,107	1,511	0,596	151,1

	61	1050	sd/1050		Log2	%
MtT22-WT	1230	420,5	2,925	1,000	0,000	100,0
MtT22-11	730,5	356,6	2,049	0,700	-0,514	70,0

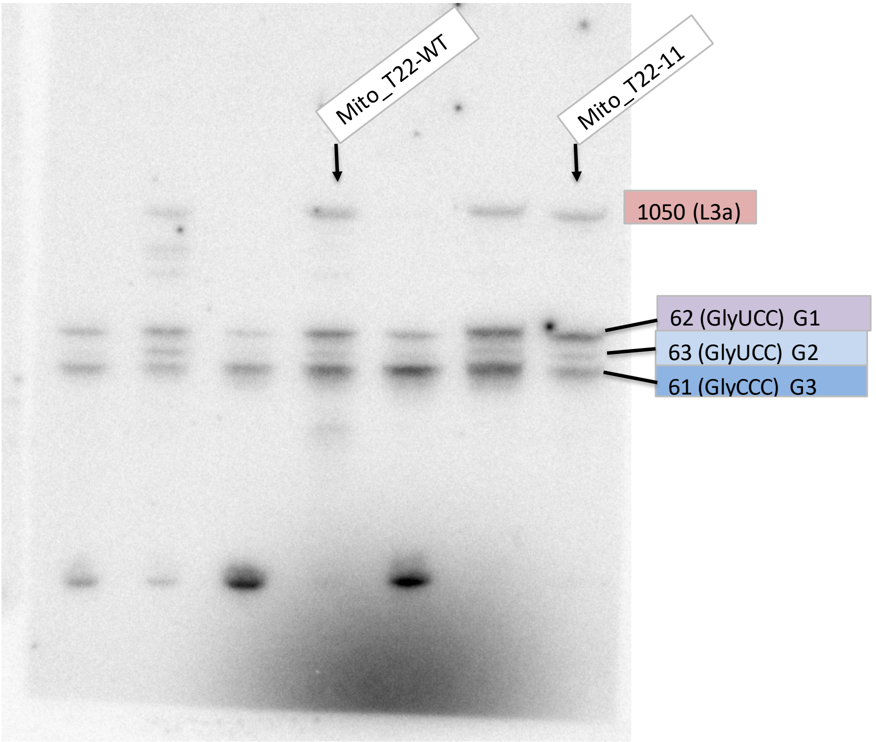

Probe_884-Bl.210611

NO.	Index	PSL	
MtT22-WT	U1	226,7	1050
MtT22-11	U2	212,3	
MtT22-WT	U3	4006	884
MtT22-11	U4	4143,5	

	884	1050	sd/1050		Log2	%
MtT22-WT	4006	226,7	17,671	1,000	0,000	100,0
MtT22-11	4143,5	212,3	19,517	1,104	0,143	110,4

Bl.21.6.11 sd884-2

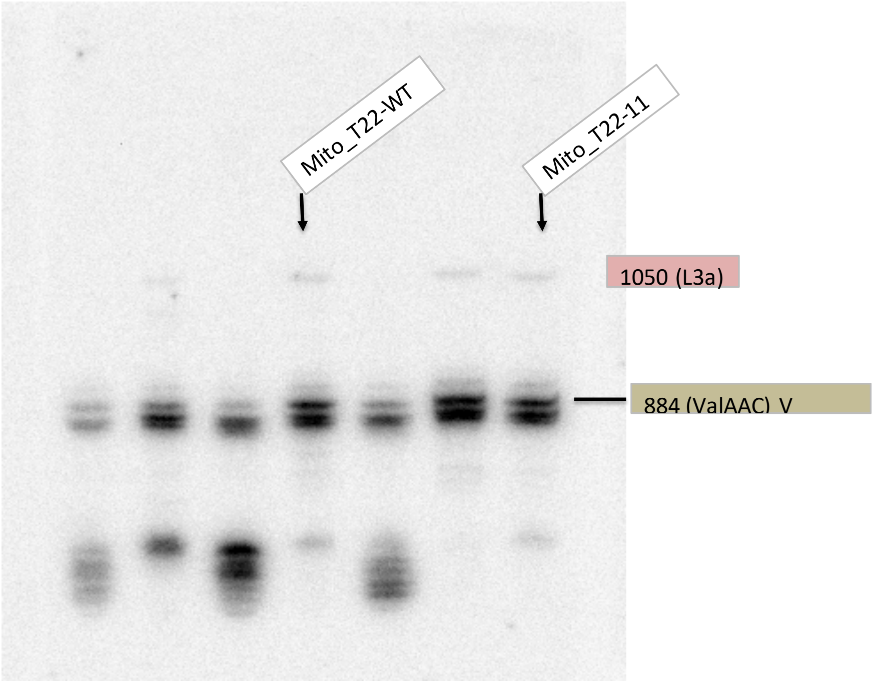

Results-Bl.210611

MtT22-11	62	831	61	63	884	AV	SD
61			72	70,0		71,0	1,3
62	130,8	126,6		151,1		136,2	13,1
63				115,7		115,7	#DIV/0!
831		116,1				116,1	#DIV/0!
884					110,4	110,4	#DIV/0!

Probe_832-Bl.220611

NO.	Index	PSL
MtT22-WT	U2	928,91
MtT22-11	U3	1140,1
MtT22-WT	U8	8822,55
MtT22-11	U9	9481,33

	832	1050	sd/1050		Log2	%
MtT22-WT	928,91	8822,55	0,105	1,000	0,000	100,0
MtT22-11	1140,1	9481,33	0,120	1,142	0,192	114,2

sd832 bl.22.6.11

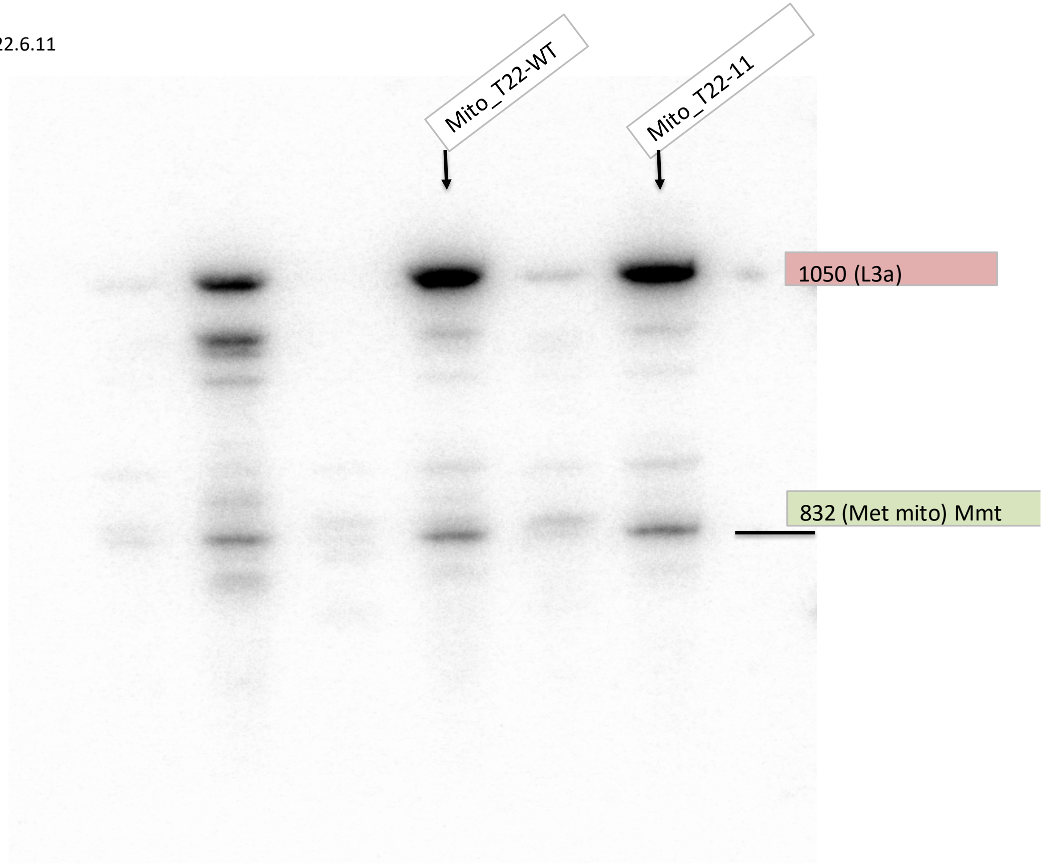

Probe_62-Bl.220611

NO.	Index	PSL
MtT22-WT	U2	6617,43
MtT22-11	U3	8150,25
MtT22-WT	U8	12843,65
MtT22-11	U9	13334,44
MtT22-WT	U14	1311,72
MtT22-11	U15	1398,04

	62	1050	sd/1050		Log2	%
MtT22-WT	6617,43	12843,65	0,515	1,000	0,000	100,0
MtT22-11	8150,25	13334,44	0,611	1,186	0,246	118,6

	832	1050	sd/1050		Log2	%
MtT22-WT	1311,72	12843,65	0,102	1,000	0,000	100,0
MtT22-11	1398,04	13334,44	0,105	1,027	0,038	102,7

sd62 bl.22.6.11

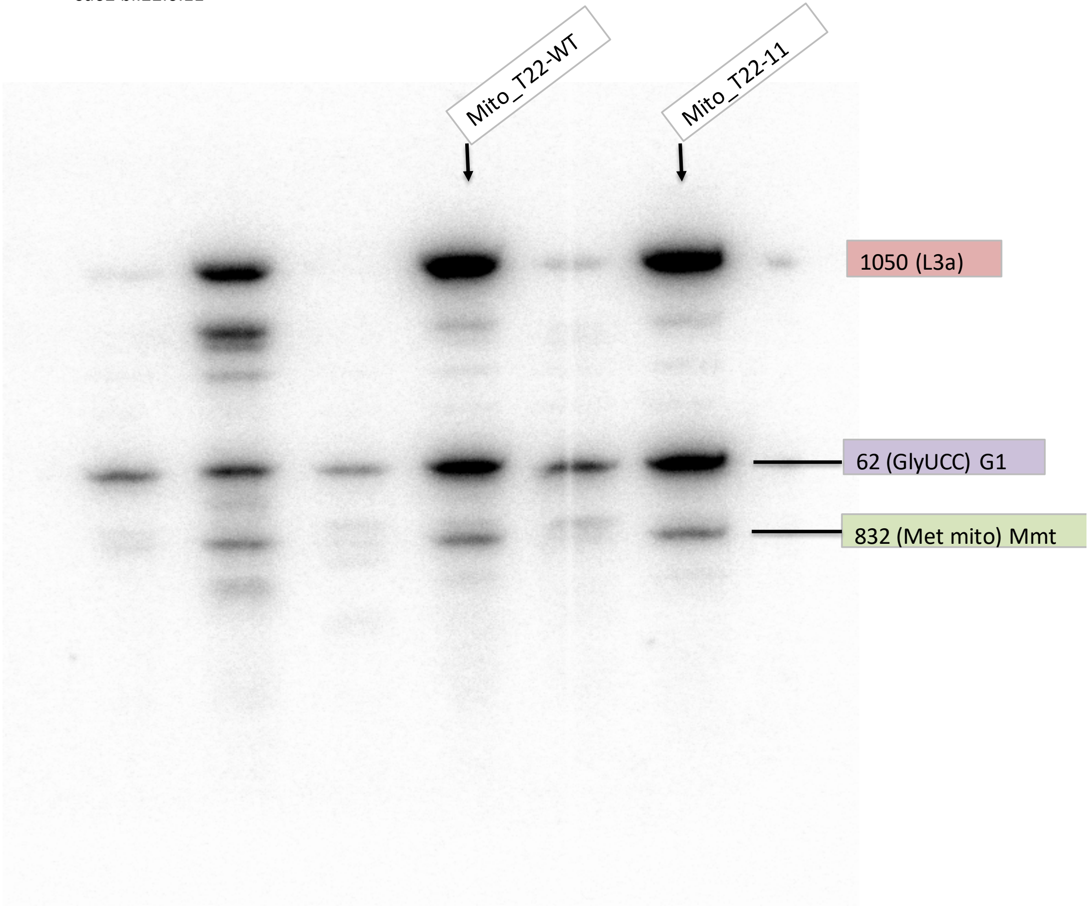

Probe_61-Bl.220611

NO.	Index	PSL	
MtT22-WT	U1	605	1050
MtT22-11	U2	587,1	
MtT22-WT	U3	2287,5	61
MtT22-11	U4	1700,2	
MtT22-WT	U5	316,3	62
MtT22-11	U6	336,5	

Bl. 22.6.11 sd61

	61	1050	sd/1050		Log2	%
MtT22-WT	2287,5	605	3,781	1,000	0,000	100,0
MtT22-11	1700,2	587,1	2,896	0,766	-0,385	77

	62	1050	sd/1050		Log2	%
MtT22-WT	316,3	605	0,523	1,000	0,000	100,0
MtT22-11	336,5	587,1	0,573	1,096	0,133	109,6

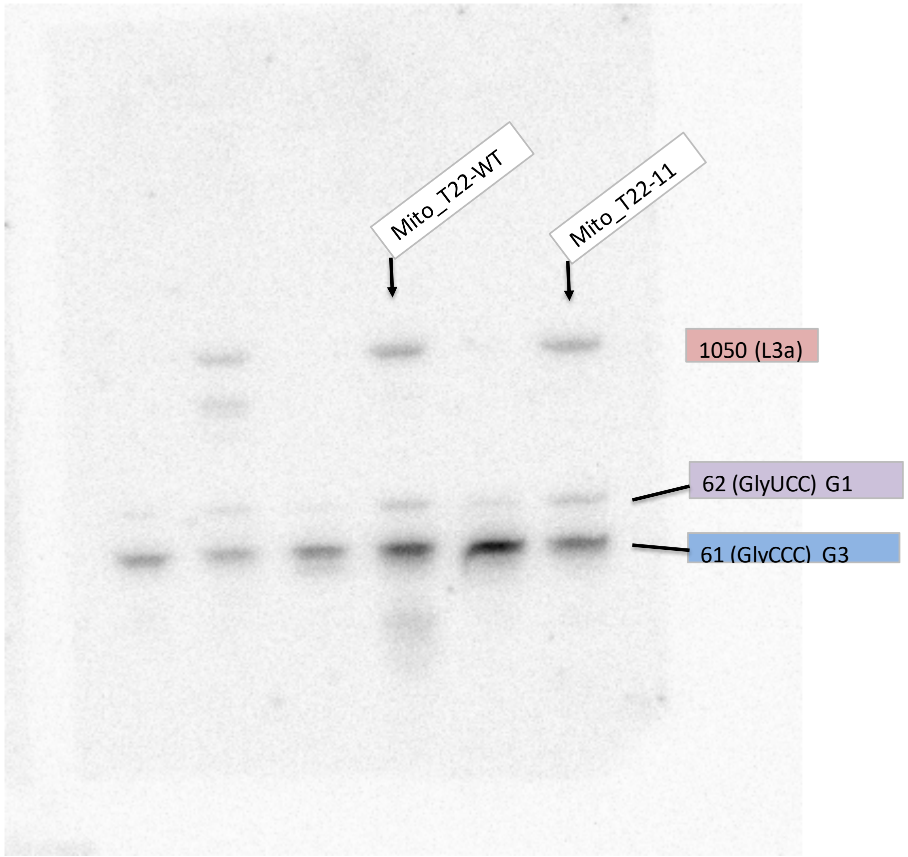

Probe_63-Bl.220611

NO.	Index	PSL	
MtT22-WT	U1	622,1	1050
MtT22-11	U2	592	
MtT22-WT	U3	594	63
MtT22-11	U4	648	
MtT22-WT	U5	1596	62
MtT22-11	U6	1938	
MtT22-WT	U7	1642	61
MtT22-11	U8	1190,3	

Bl.22.6.11 sd63

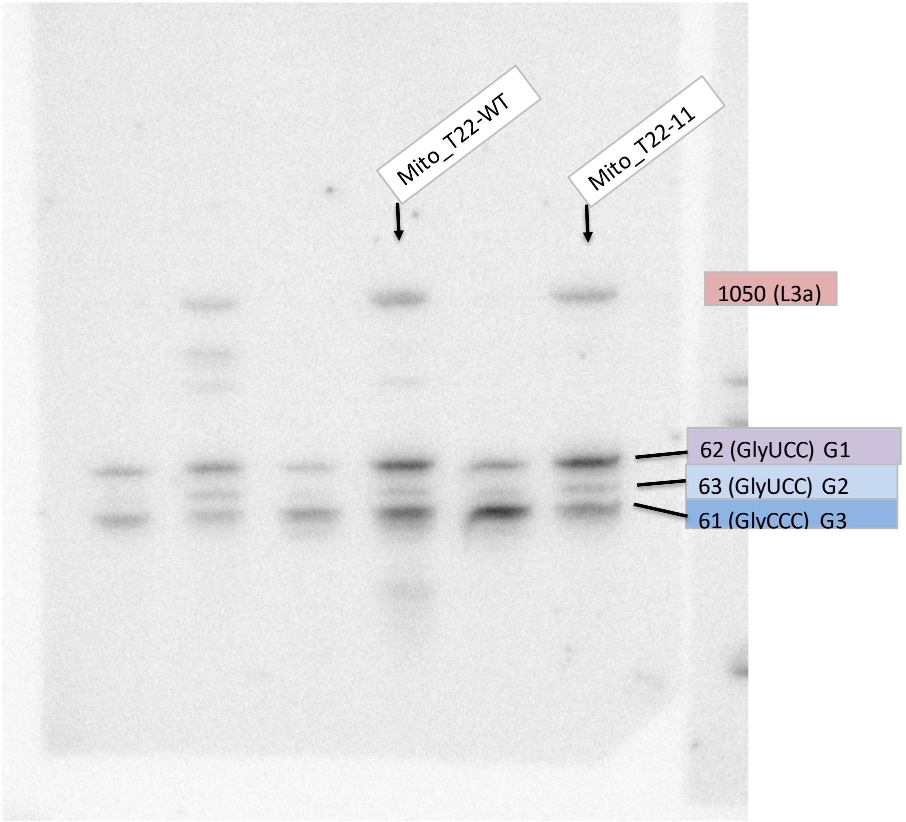

	63	1050	sd/1050		Log2	%
MtT22-WT	594	622,1	0,955	1,000	0,000	100,0
MtT22-11	648	592	1,095	1,146	0,197	114,6

	62	1050	sd/1050		Log2	%
MtT22-WT	1596	622,1	2,566	1,000	0,000	100,0
MtT22-11	1938	592	3,274	1,276	0,352	127,6

	61	1050	sd/1050		Log2	%
MtT22-WT	1642	622,1	2,639	1,000	0,000	100,0
MtT22-11	1190,3	592	2,011	0,762	-0,393	76,2

Results-Bl.220611

MtT22-11	832	62	61	63		AV	SD
61			76,6	76,2		76,4	0,3
62		118,6	109,6	127,6		118,6	9,0
63				114,6		114,6	#DIV/0!
832	114,2	102,7				108,4	8,2

Sum up_Fig5B

		GlyGCC	GlyUCC	GlyCCC	ValAAC	Leu AAG	Met mt	Gln mt	L3a
MtT22-8	Probe	62	63	61	884	958	832	831	1050
	Name	G1	G2	G3	V	L	Mmt	Qmt	
**21.6.2011**	100	130,8	115,7	72	110,4			116,1	
**22.6.2011**	100	118,6	114,6	77			114,2		100,0
**2.9.11**	100	100,7	132,6	62,2	92,3	76,5	101,4	95,5	100,0
**8.9.11(1rs)**	100				115,60	95,45			100
**8.9.11(3ms)**	100				92,83	77,51	115,10	85,30	100
AV		117	121	70	103	83	110	99	100
SD		15	10	8	12	11	8	16	0
TTEST		**0,10**	**0,03**	**0,01**	**0,34**	**0,06**	**0,07**	**0,46**	

G1	G2	G3	V	L	Mmt	Qmt
117	121	70	103	83	110	99
15	10	8	12	11	8	16

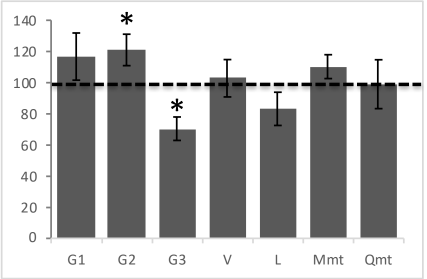

* > 0,05
